# Publisher Correction: A DHODH inhibitor increases p53 synthesis and enhances tumor cell killing by p53 degradation blockage

**DOI:** 10.1038/s41467-023-40764-2

**Published:** 2023-08-18

**Authors:** Marcus J. G. W. Ladds, Ingeborg M. M. van Leeuwen, Catherine J. Drummond, Su Chu, Alan R. Healy, Gergana Popova, Andrés Pastor Fernández, Tanzina Mollick, Suhas Darekar, Saikiran K. Sedimbi, Marta Nekulova, Marijke C. C. Sachweh, Johanna Campbell, Maureen Higgins, Chloe Tuck, Mihaela Popa, Mireia Mayoral Safont, Pascal Gelebart, Zinayida Fandalyuk, Alastair M. Thompson, Richard Svensson, Anna-Lena Gustavsson, Lars Johansson, Katarina Färnegårdh, Ulrika Yngve, Aljona Saleh, Martin Haraldsson, Agathe C. A. D’Hollander, Marcela Franco, Yan Zhao, Maria Håkansson, Björn Walse, Karin Larsson, Emma M. Peat, Vicent Pelechano, John Lunec, Borivoj Vojtesek, Mar Carmena, William C. Earnshaw, Anna R. McCarthy, Nicholas J. Westwood, Marie Arsenian-Henriksson, David P. Lane, Ravi Bhatia, Emmet McCormack, Sonia Laín

**Affiliations:** 1 Department of Microbiology, Tumor and Cell Biology (MTC), Karolinska Institutet, SE-171 77 Stockholm, Sweden; 2 SciLifeLab, Department of Microbiology, Tumor and Cell Biology (MTC), Karolinska Institutet, Tomtebodavägen 23, SE-171 21 Stockholm, Sweden; 3 Division of Hematology and Oncology, Comprehensive Cancer Center, 1720 2nd Avenue South, NP2540, Birmingham, AL 35294-3300 USA; 4 School of Chemistry and Biomedical Sciences Research Complex, University of St. Andrews and EaStCHEM, St. Andrews, Fife, Scotland KY16 9ST UK; 5 RECAMO, Masaryk Memorial Cancer Institute, Zluty Kopec 7, 65653 Brno, Czech Republic; 6 Centre for Oncology and Molecular Medicine, University of Dundee, Ninewells Hospital and Medical School, Dundee, Tayside DD1 9SY UK; 7 Centre for Cancer Biomarkers, CCBIO, Department of Clinical Science, Hematology Section, University of Bergen, 5021 Bergen, Norway; 8 Department of Breast Surgical Oncology, MD Anderson Cancer Center, Holcombe Boulevard, Houston, TX 77030 USA; 9 Department of Pharmacy, Uppsala University Drug Optimization and Pharmaceutical Profiling Platform (UDOPP), Department of Pharmacy, Uppsala University, SE-752 37 Uppsala, Sweden; 10 Chemical Biology Consortium Sweden, Science for Life Laboratory, Division of Translational Medicine and Chemical Biology, Department of Medical Biochemistry and Biophysics, Karolinska Institutet, SE-171 21 Stockholm, Sweden; 11 Drug Discovery and Development Platform, Science for Life Laboratory, Tomtebodavägen 23, SE-171 21 Solna, Sweden; 12 Department of Medicinal Chemistry, Science for Life Laboratories, Uppsala University, SE-751 23 Uppsala, Sweden; 13 Newcastle Cancer Centre, Northern Institute for Cancer Research, Newcastle University, Newcastle, NE1 7RU UK; 14 SARomics Biostructures, Medicon Village, SE-223 81 Lund, Sweden; 15 The Wellcome Trust Centre for Cell Biology, Institute of Cell Biology, University of Edinburgh, Edinburgh, EH9 3JR UK; 16 Department of Medicine, Haematology Section, Haukeland University Hospital, Bergen, Norway

Correction to: *Nature Communications* 10.1038/s41467-018-03441-3, published online 16 March 2018

This Article contains an error in Fig. 6. The submitted and peer reviewed versions of this article contain distinct distributions of sub-G1 cells in Fig 6 d, however, one of those distributions was inadvertently duplicated during the production process. Additionally, one of the distributions in Fig 6 d was omitted during the production process.

The correct version of Fig 6 is:
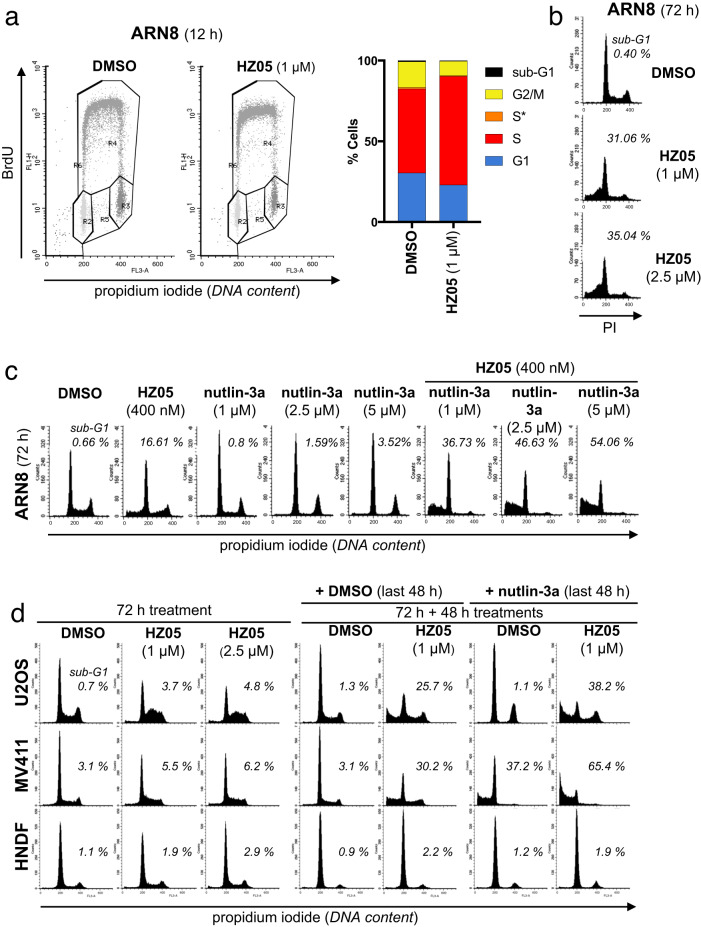


which replaces the previous incorrect version of Fig. 6. The error has been corrected in the PDF or HTML version of the Article.

