# Supplementary material for: A DHODH inhibitor increases p53 synthesis and enhances tumor cell killing by p53 degradation blockage
Source: Nat Commun. 2018 Mar 16;9:1107. doi: 10.1038/s41467-018-03441-3 (PMC5856786; doi:10.1038/s41467-018-03441-3)
Supplement: Supplementary file 1 — Supplementary Information [file 41467_2018_3441_MOESM1_ESM.pdf]

A DHODH inhibitor increases p53 synthesis and enhances tumor cell killing by p53 degradation blockage

Supplementary Information

Marcus J.G.W. Ladds[1,16#], Ingeborg M.M. van Leeuwen[1#], Catherine J. Drummond[1#], Su Chu[2], Alan R. Healy[3], Gergana Popova[1], Andrés Pastor-Fernández[1], Tanzina Mollick[1,16], Suhas Darekar[1,16], Saikiran K. Sedimbi[1,16], Marta Nekulova[1,15], Marijke C.C. Sachweh[1], Johanna Campbell[4], Maureen Higgins[4], Chloe Tuck[1], Mihaela Popa[5], Mireia Mayoral Safont[5], Pascal Gelebart[5], Zinayida Fandalyuk[5], Alastair M. Thompson[6], Richard Svensson[7], Anna-Lena Gustavsson[8], Lars Johansson[8], Katarina Färnegårdh[9], Ulrika Yngve[10], Aljona Saleh[10], Martin Haraldsson[9], Agathe C.A. D'Hollander[3], Marcela Franco[1], Yan Zhao[11], Maria Håkansson[14], Björn Walse[14], Karin Larsson[1], Emma M. Peat[12], Vicent Pelechano[16], John Lunec[11], Borivoj Vojtesek[15], Mar Carmena[12], William C. Earnshaw[12], Anna R. McCarthy[1], Nicholas J. Westwood[3], Marie Arsenian Henriksson[1], David P. Lane[1,16], Ravi Bhatia[2], Emmet McCormack[5,13] and Sonia Laín[1,16**]*

[1] Department of Microbiology, Tumor and Cell Biology (MTC), Karolinska Institutet, SE-171 77, Stockholm, Sweden

[2] Division of Hematology & Oncology, Comprehensive Cancer Center, 1720 2nd Avenue South, NP2540, Birmingham, AL 35294-3300, USA

[3] School of Chemistry and Biomedical Sciences Research Complex, University of St. Andrews and EaStCHEM, St. Andrews, Fife, Scotland, KY16 9ST, United Kingdom

[4] Centre for Oncology and Molecular Medicine, University of Dundee, Ninewells Hospital and Medical School, Dundee, Tayside, DD1 9SY, UK

[5] Centre for Cancer Biomarkers, CCBIO, Department of Clinical Science, Hematology Section, University of Bergen, 5021 Bergen, Norway

[6] Department of Breast Surgical Oncology, MD Anderson Cancer Center, Holcombe Boulevard, Houston 77030, USA

[7] Department of Pharmacy, Uppsala University Drug Optimization and Pharmaceutical Profiling Platform (UDOPP), Department of Pharmacy, Uppsala University, SE-752 37, Uppsala, Sweden

[8] Chemical Biology Consortium Sweden, Science for Life Laboratory, Division of Translational Medicine and Chemical Biology, Department of Medical Biochemistry and Biophysics, Karolinska Institutet, SE-171 21 Stockholm, Sweden

[9] Drug Discovery and Development Platform, Science for Life Laboratory, Tomtebodavägen 23, SE-171 21 Solna, Sweden.

[10] Department of Medicinal Chemistry, Science for Life Laboratories, Uppsala University, SE-751 23 Uppsala, Sweden.

[11] Newcastle Cancer Centre, Northern Institute for Cancer Research, Newcastle University, Newcastle, NE1 7RU, UK

[12] The Wellcome Trust Centre for Cell Biology, Institute of Cell Biology, University of Edinburgh, Edinburgh EH9 3JR, UK

[13] Department of Medicine, Haematology Section, Haukeland University Hospital, Bergen, Norway

[14] SARomics Biostructures, Medicon Village, SE-223 81, Lund, Sweden

[15] RECAMO, Masaryk Memorial Cancer Institute, Zluty Kopec 7, 65653 Brno, Czech Republic

[16] SciLifeLab, Department of Microbiology, Tumor and Cell Biology (MTC), Karolinska Institutet, Tomtebodavägen 23, SE-171 21, Stockholm, Sweden.

# these authors contributed equally

** deceased*

*** corresponding author*

Supplementary Methods

Chemicals and reagents were obtained from commercial suppliers and were used as received unless otherwise stated. Analogues to *rac*-HZ00 were obtained from Chembridge, and the purity was verified by LCMS to be 95 % (*rac*-HZ02), 93 % (*rac*-HZ05) and 84 % (*rac*-HZ25). All reactions involving moisture sensitive reagents were performed in oven or flame dried glassware under a positive pressure of nitrogen. Tetrahydrofuran (THF) and dichloromethane (DCM) were obtained dry from a solvent purification system (MBraun, SPS-800). Thin-layer chromatography was performed using glass plates coated with silica gel (with fluorescent indicator UV_{254}). Developed plates were air-dried and analysed under a UV lamp or by $KMnO_4$ dip staining. Flash column chromatography was performed using silica gel (40–63 μm). Melting points were recorded in open capillaries using an Electrothermal 9100 melting point apparatus. Values are quoted to the nearest 1 °C and are uncorrected. Infrared spectra were recorded on a Perkin Elmer Spectrum GX FT-IR spectrometer using KBr discs (KBr) as stated. Absorption maxima are reported as wavenumbers (cm^{-1}). Low resolution (LR) and high resolution (HR) electrospray mass spectral (ES-MS) analyses were acquired by electrospray ionisation (ESI), electron impact (EI) or chemical ionisation (CI). These were acquired by the EPSRC National Mass Spectrometry Service or within the School of Chemistry, University of St Andrews. Nuclear magnetic resonance (NMR) spectra were acquired on either a Bruker Avance 300 (^{1}H, 300.1 MHz; ^{13}C, 75.5 MHz), Bruker Avance II 400 (^{1}H, 400.1 MHz; ^{13}C, 100.6 MHz), Bruker Avance 500 (^{1}H, 499.9 MHz; ^{13}C, 125.7 MHz) or Bruker Avance III 500 (^{1}H, 500.1 MHz, ^{13}C, 125.7 MHz) spectrometer and in the deuterated solvent stated. All NMR spectra were acquired using the deuterated solvent as the lock. Coupling constants (J) are quoted in Hz and are recorded to the nearest 0.1 Hz. The following abbreviations are used; s, singlet; d, doublet; dd, doublet of doublets; t, triplet; m, multiplet; q, quartet; and br, broad. HPLC analyses were obtained on a Gilson HPLC consisting of a Gilson 305 pump, Gilson 306 pump, Gilson 811C dynamic mixer, Gilson 805 manometric module, Gilson 401C dilutor, Gilson 213XL sample injector and sample detection was performed with a Gilson 118 UV/vis detector. Separation was achieved using a Chiralpak AD-H column. Optical rotations were measured on a Perkin Elmer Precisely/Model-341 polarimeter operating at the sodium D line with a 100 mm path cell.

Experimental Procedures

The reaction scheme for the following syntheses has been detailed in supplementary figure 13a and b.

1-(2-Fluorophenyl)-6,7-dihydro-1*H*-indazol-4(5*H*)-one (1). The compound was prepared according to procedures described in the literature for the synthesis of molecules similar to **1**[1-2]. mp 104–106 °C; IR (KBr) ν_{max}: 2957, 1663 (C=O), 1508, 1408, 1226; ^{1}H NMR (400 MHz, $CDCl_3$) δ 8.10 (s, 1H, C3-H), 7.60–7.43 (m, 2H, 2 × ArH), 7.36–7.22 (m, 2H, 2 × ArH), 2.82–2.78 (m, 2H, C7-H_2), 2.57–2.53 (m, 2H, C5-H_2), 2.27–2.06 (m, 2H, C6-H_2). ^{1}H NMR data has been detailed in supplementary figure 14b. ^{13}C NMR (101 MHz, $CDCl_3$) δ 193.2 (CO), 157.2 (C), 154.7 (C), 151.5 (C7a), 139.0 (C3), 130.8 (d, J = 7.7 Hz, CH), 128.4 (CH), 125.1 (d, J = 3.9 Hz, CH), 120.1 (C3a), 116.8 (d, J = 19.7 Hz, CH), 37.9 (C5), 23.4 (C6), 22.0 (C7). ^{13}C NMR data has been detailed in supplementary figure 14c. *m/z* (ES^+) 252.89 ($[M + Na]^+$, 100 %); HRMS (ES^+) Calcd for $C_{13}H_{11}N_2OFNa$ $[M + Na]^+$: 253.0753, found 253.0757.

***N*-(1-(2-Fluorophenyl)-4,5,6,7-tetrahydro-1*H*-indazol-4-yl)picolinamide (*rac*-HZ00).** Following the procedure described[1], a solution of **1** (1.0 g, 4.3 mmol, 1.0 equiv) in 2-propanol (80 mL) was treated, under vigorous stirring, with ammonium acetate (3.3 g, 43.4 mmol, 10.0 equiv). After complete dissolution, molecular sieves (4 Å, 1.5 g) and $NaBH_3CN$ (1.3 g, 21.7 mmol, 5.0 equiv) were added and the reaction mixture was stirred for 12 h at 70 °C. The solution was concentrated *in vacuo*; the residue was diluted with EtOAc (200 mL) and washed thoroughly with a 2 M aqueous solution of NaOH (20 mL) and brine (20 mL), dried over $MgSO_4$, filtered and concentrated. Without further purification, the

crude intermediate 1-(2-fluorophenyl)-4,5,6,7-tetrahydro-1*H*-indazol-4-amine (*rac*-**3**) was directly used in the next step. To a solution of 2-picolinic acid (0.48 g, 3.9 mmol, 2.0 equiv), HOBt (0.79 g, 5.8 mmol, 1.5 equiv), EDC·HCl (1.12 g, 5.8 mmol, 1.5 equiv) and Et_3N (0.21 mL, 5.8 mmol, 1.5 equiv) in DCM (80 mL) were added *rac*-**3** (0.90 g, 3.9 mmol, 1.0 equiv) and DMAP (48 mg, 0.39 mmol, 0.1 equiv). The resulting solution was stirred at RT overnight. The solution was concentrated *in vacuo*; the residue was diluted with EtOAc (40 mL) and washed thoroughly with a sat. aq. $NaHCO_3$ solution (20 mL) and brine (20 mL), dried over $MgSO_4$, filtered and concentrated. Purified *via* the Biotage SP4 (silica-packed SNAP column 10 g; 20–50 % EtOAc/hexanes) to give *rac*-**HZ00** as a white solid (0.92 g, 65 % over 2 steps). mp 96–98 °C; IR (KBr) ν_{max}: 3288 (NH), 2945, 1661 (C=O), 1516; ^{1}H NMR (500 MHz, $CDCl_3$) δ 8.53 (ddd, *J* = 4.8, 1.7, 0.9 Hz, 1H, C6'-H), 8.34–8.15 (m, 2H, NH, C3'-H), 7.87 (td, *J* = 7.7, 1.7 Hz, 1H, C4'-H), 7.69 (s, 1H, C3-H), 7.57–7.35 (m, 3H, 2 × ArH, C5'-H), 7.34–7.15 (m, 2H, 2 × ArH), 5.35 (dt, *J* = 8.3, 5.6 Hz, 1H, C4-H), 2.73–2.48 (m, 2H, C7-H_2), 2.27–2.09 (m, 1H, C5-H), 2.03–1.85 (m, 3H, C5-H, C6-H_2). ^{1}H NMR data has been detailed in supplementary figure 15b. ^{13}C NMR (126 MHz, $CDCl_3$) δ 163.9 (CO), 156.5 (d, *J* = 251.5 Hz, C), 150.1 (C2'), 148.2 (C6'), 141.9 (C7a), 139.5 (C3), 137.5 (C4'), 130.1 (d, *J* = 7.8 Hz, CH), 128.8 (CH), 127.5 (d, *J* = 11.7 Hz, C), 126.3 (C5'), 124.9 (d, *J* = 3.7 Hz, CH), 122.4 (C3'), 118.1 (C3a), 116.8 (d, *J* = 20.0 Hz, CH), 42.5 (C4), 30.3 (C5), 21.7 (C7), 20.3 (C6). ^{13}C NMR data has been detailed in supplementary figure 15c. *m/z* (ES^+) 358.86 ($[M + Na]^+$, 100 %); HRMS (ES^+) Calcd for $C_{19}H_{17}N_4OFNa$ $[M + Na]^+$: 359.1284, found 359.1283.

(*S*)-*N*-((*R*)-1-(2-Fluorophenyl)-4,5,6,7-tetrahydro-1*H*-indazol-4-yl)-2-methylpropane-2-sulfinamide ((*S*,*R*)-2). To (S_S)-2-methyl-2-propanesulfinamide (2.44 g, 20.1 mmol, 1.0 equiv) and $Ti(OEt)_4$ (8.4 mL, 40.2 mmol, 2.0 equiv) in THF (40 mL) at RT was added **1** (4.64 g, 20.1 mmol, 1.0 equiv). The mixture was heated at 75 °C for 12 h. The mixture was then cooled to –48 °C and L-Selectride® in THF (60.3 mL, 1 M, 3.0 equiv) was added dropwise. Once the reduction was complete, the reaction mixture was warmed to 0 °C and MeOH was added dropwise until gas evolution was no longer observed. The crude reaction mixture was poured into brine (20 mL) whilst being vigorously stirred. The resulting suspension was filtered through celite, and the filter cake was washed with EtOAc (2 × 10 mL). The filtrate was washed with brine (30 mL), and the brine layer was back extracted with EtOAc (3 × 20 mL). The combined organic extracts were dried over $MgSO_4$, filtered, concentrated *in vacuo* and purified *via* the Biotage SP4 (silica-packed SNAP column 180 g; 20–70 % EtOAc/hexanes) to give the title product (*S*,*R*)-**2** as a white solid (4.89 g, 73 %). A sample of (*S*,*R*)-**2** suitable for X-ray crystallographic analysis was prepared by recrystallization from DCM which confirmed the absolute configuration to be as drawn; mp 112–114 °C; IR (KBr) ν_{max}: 3433 (NH), 3201, 2963, 1514, 1031; ^{1}H NMR (500 MHz, $CDCl_3$) δ 7.65 (s, 1H, CH), 7.47–7.36 (m, 2H, 2 × ArH), 7.26–7.19 (m, 2H, 2 × ArH), 4.53–4.49 (m, 1H, CH), 3.37 (d, *J* = 9.4 Hz, 1H, NH), 2.59–2.44 (m, 2H, CH_2), 2.34–2.23 (m, 1H, CH), 1.95 (m, 1H, CH), 1.84 (m, 2H, CH, CH), 1.26 (s, 9H, 3 × CH_3). ^{1}H NMR data has been detailed in supplementary figure 16b. ^{13}C NMR (101 MHz, $CDCl_3$) δ 157.7 (C), 155.2 (C), 141.5 (C), 139.4 (CH), 130.1 (d, *J* = 7.8 Hz, CH), 128.7 (CH), 124.9 (d, *J* = 4.0 Hz, CH), 119.2 (C), 116.8 (d, *J* = 19.9 Hz, CH), 56.3 (C), 50.8 (CH), 33.3 (CH_2), 22.9 (3 × CH_3), 21.6 (CH_2), 20.3 (CH_2). ^{13}C NMR data has been detailed in supplementary figure 16c. *m/z* (ES^+) 357.84 ($[M + Na]^+$, 100 %); HRMS (ES^+) Calcd for $C_{17}H_{23}N_3OSF$ $[M + H]^+$: 358.1365, found 358.1366.

(4*R*)-1-(2-Fluorophenyl)-4,5,6,7-tetrahydro-1*H*-indazol-4-amine ((*R*)-3). Concentrated HCl (4 mL) was added dropwise to a solution of (*S*,*R*)-**2** (1.4 g, 4.2 mmol) in methanol (40 mL), and the solution was stirred at RT for 4 h. The reaction was quenched with a sat. aq. $NaHCO_3$ solution (10 mL) and diluted with DCM (20 mL) and water (20 mL). The organic layer was washed with a sat. aq. $NaHCO_3$ solution (20 mL) and then brine (20 mL), dried with $MgSO_4$, filtered and concentrated *in vacuo* to give the free amine (*R*)-**3**: ^{1}H NMR (400 MHz, $CDCl_3$) δ 7.72 (s, 1H, CH), 7.53–7.32 (m, 2H, 2 × ArH), 7.31–7.09 (m, 2H, 2 × ArH), 4.11–4.00 (m, 1H, CH), 2.65–2.33 (m, 2H, CH_2), 2.16–1.46 (m, 4H, 2 × CH_2); ^{13}C NMR (101 MHz, $CDCl_3$) δ 157.6 (C), 155.1 (C), 140.6 (C), 138.9 (CH), 129.8 (d, *J* = 7.8 Hz, CH), 128.6 (CH), 124.7 (d, *J* = 3.8 Hz, CH), 122.0 (C), 116.6 (d, *J* = 20.1 Hz, CH), 44.6 (CH), 34.1

(CH_2), 21.7 (CH_2), 20.2 (CH_2); *m/z* (ES^+) 231.09 ($[M+H]^+$, 100 %). Without further purification, the crude (*R*)-**3** was used directly in the next step.

(*R*)-*N*-(1-(2-Fluorophenyl)-4,5,6,7-tetrahydro-1*H*-indazol-4-yl)picolinamide ((*R*)-HZ00). To a solution of 2-picolinic acid (0.91 g, 7.44 mmol, 2.0 equiv), HOBt (0.75 g, 5.58 mmol, 1.5 equiv), EDC·HCl (1.07 g, 5.58 mmol, 1.5 equiv) and Et_3N (0.78 mL, 5.58 mmol, 1.5 equiv) in DCM (75 mL) were added (*R*)-**3** (0.86 g, 3.72 mmol, 1.0 equiv) and DMAP (45 mg, 0.37 mmol, 0.1 equiv). The resulting solution was stirred at RT overnight. The solution was concentrated *in vacuo*. The residue was then diluted with EtOAc (30 mL) and washed thoroughly with a sat. aq. $NaHCO_3$ solution (20 mL) and brine (20 mL), dried over $MgSO_4$, filtered and concentrated *in vacuo*. The resulting solid was purified using a Biotage SP4 (silica-packed SNAP column 100 g; 20–50 % EtOAc/hexanes) to give (*R*)-**HZ00** as a white solid (1.16 g, 82 % over 2 steps); mp 117–119 °C; $[\alpha]_D^{20}$ = +71.3 (*c* 0.1, $CHCl_3$); Chiral HPLC analysis Chiralpak AD-H (5 % IPA/hexane, 1 mL min^{-1}, 254 nm, 30 °C) t_R (major) 31.3 min, t_R (minor) 35.9 min, 98 % ee; IR (KBr) ν_{max}: 3443 (NH), 3291, 2946, 1661 (C=O), 1530; ^1H NMR (500 MHz, $CDCl_3$) δ 8.53 (ddd, *J* = 4.7, 1.7, 0.9 Hz, 1H, C<u>H</u>), 8.34–8.20 (m, 2H, N<u>H</u>, C<u>H</u>), 7.87 (td, *J* = 7.7, 1.7 Hz, 1H, C<u>H</u>), 7.69 (s, 1H, C<u>H</u>), 7.51–7.35 (m, 3H, 2 × Ar<u>H</u>, C<u>H</u>), 7.32–7.11 (m, 2H, 2 × Ar<u>H</u>), 5.36 (dd, *J* = 8.6. 4.8 Hz, 1H, C<u>H</u>), 2.74–2.48 (m, 2H, C<u>H</u>$_2$), 2.30–2.07 (m, 1H, C<u>H</u>), 2.07–1.74 (m, 3H, C<u>H</u>, C<u>H</u>$_2$). ^1H NMR data has been detailed in supplementary figure 17b. ^{13}C NMR (126 MHz, $CDCl_3$) δ 163.8 (CO), 156.4 (d, *J* = 251.5 Hz, C), 150.0 (C), 148.2 (CH), 141.9 (C), 139.5 (CH), 137.5 (CH), 130.1 (d, *J* = 7.7 Hz, CH), 128.7 (CH), 127.5 (d, *J* = 11.8 Hz, C), 126.3 (CH), 124.9 (d, *J* = 3.9 Hz, CH), 122.5 (CH), 118.1 (C), 116.8 (d, *J* = 20.1 Hz, CH), 42.5 (CH), 30.3 (CH_2), 21.7 (CH_2), 20.2 (CH_2). ^{13}C NMR data has been detailed in supplementary figure 17c. *m/z* (ES^+) 359.12 ($[M + Na]^+$, 100 %); HRMS (ES^+) Calcd for $C_{19}H_{18}N_4ONa$ $[M + Na]^+$: 359.1284, found 359.1281.

(*S*)-N-((*S*)-1-(2-Fluorophenyl)-4,5,6,7-tetrahydro-1*H*-indazol-4-yl)-2-methylpropane-2-sulfinamide ((*S*,*S*)-2). To (S_S)-2-methyl-2-propanesulfinamide (316 mg, 2.6 mmol, 1.2 equiv) and $Ti(OEt)_4$ (0.91 mL, 4.3 mmol, 2.0 equiv) in THF (5 mL) at RT was added **1** (500 mg, 2.2 mmol, 1.0 equiv). The mixture was heated at 75 ℃ for 12 h. The mixture was cooled to –48 ℃ and $NaBH_4$ (205 mg, 5.4 mmol, 2.5 equiv) was added. Once the reduction was complete, the reaction mixture was warmed to 0 ℃ and MeOH was added dropwise until gas evolution was no longer observed. The crude reaction mixture was poured into brine (5 mL) whilst being vigorously stirred. The resulting suspension was filtered through a celite, and the filter cake was washed with EtOAc (2 × 5 mL). The filtrate was washed with brine (20 mL), and the brine layer was extracted with EtOAc (3 × 10 mL). The combined organic extracts were dried over $MgSO_4$, filtered, concentrated *in vacuo* and purified *via* the Biotage SP4 (silica-packed SNAP column 10 g; 20–70 % EtOAc/hexanes) to give the title product (*S*,*S*)-**2** as a white solid (577 mg, 79 %). mp 94–96 ℃; IR (KBr) ν_{max}: 3418 (NH), 3220, 2921, 1667, 1460, 1194; ^1H NMR (500 MHz, $CDCl_3$) δ 7.81 (s, 1H, C3-<u>H</u>), 7.46 (td, *J* = 7.8, 1.7 Hz, 1H, Ar<u>H</u>), 7.39 (tdd, *J* = 7.8, 4.8, 1.7 Hz, 1H, Ar<u>H</u>), 7.33–7.16 (m, 2H, 2 × Ar<u>H</u>), 4.59 (q, *J* = 4.9 Hz, 1H, C4-<u>H</u>), 3.31 (d, J = 4.9 Hz, 1H, N<u>H</u>), 2.52 (qt, *J* = 17.0, 6.1 Hz, 1H, C7-<u>H</u>$_2$), 2.00–1.91 (m, 2H, C5-<u>H</u>, C6-<u>H</u>), 1.91–1.72 (m, 2H, C5-<u>H</u>, C6-<u>H</u>), 1.24 (s, 9H, 3 × C<u>H</u>$_3$). All ^1H NMR data is displayed in supplementary figure 18b. ^{13}C NMR (101 MHz, $CDCl_3$) δ 156.4 (d, *J* = 251.4 Hz, C), 141.8 (C7a), 139.8 (C3), 130.1 (d, *J* = 7.8 Hz, CH), 128.8 (CH), 127.5 (d, *J* = 11.9 Hz, C), 124.9 (d, *J* = 3.8 Hz, CH), 118.9 (C3a), 116.7 (d, *J* = 20.0 Hz, CH), 55.7 (C), 48.3 (C4), 31.5 (C5), 22.8 (3 × CH_3), 21.7 (C7), 19.4 (C8). All ^{13}C NMR data is displayed in supplementary figure 18c. m/z (ES^+) 358.98 ($[M + Na]^+$, 100 %).

(4*S*)-1-(2-Fluorophenyl)-4,5,6,7-tetrahydro-1*H*-indazol-4-amine ((*S*)-3). Concentrated HCl (0.4 mL) was added dropwise to a solution of (*S*,*S*)-**2** (136 mg, 0.41 mmol, 1.0 equiv) in methanol (5 mL), and the solution was stirred at RT for 4 h. The reaction was quenched with a sat. aq. $NaHCO_3$ solution (5 mL), and diluted with DCM (5 mL) and water (5 mL). The organic layer was washed with a sat. aq. $NaHCO_3$ solution (5 mL) and brine (5 mL), dried over $MgSO_4$, filtered and concentrated *in vacuo* to give crude (*S*)-**3**. ^1H NMR (400 MHz, $CDCl_3$) δ 7.71 (s, 1H, C3-<u>H</u>), 7.50–7.34 (m, 2H, 2 × Ar<u>H</u>), 7.28–7.17 (m, 2H, 2 × Ar<u>H</u>), 4.05–4.00 (m, 1H, C4-<u>H</u>), 2.63–2.41 (m, 2H, C7-<u>H</u>$_2$), 2.13–1.48 (m, 4H, C5-<u>H</u>$_2$,

C6-H̲$_2$); ^{13}C NMR (101 MHz, $CDCl_3$) δ 157.5 (C), 155.0 (C), 140.6 (C7a), 138.2 (C3), 129.8 (d, J = 7.7 Hz, CH), 128.6 (CH), 124.6 (d, J = 3.8 Hz, CH), 121.9 (C3a), 116.6 (d, J = 20.3 Hz, CH), 44.6 (C4), 34.1 (C5), 21.6 (C7), 20.2 (C6); *m/z* (ES^+) 232.11 ($[M+H]^+$, 100 %). Without further purification, crude (*S*)-3 was used directly in the next step.

(*S*)-*N*-(1-(2-Fluorophenyl)-4,5,6,7-tetrahydro-1*H*-indazol-4-yl)picolinamide ((*S*)-HZ00). To a solution of 2-picolinic acid (100 mg, 0.81 mmol, 2.0 equiv), HOBt (83 mg, 0.62 mmol, 1.5 equiv), EDC·HCl (119 mg, 0.62 mmol, 1.5 equiv) and Et_3N (0.17 mL, 1.23 mmol, 3.0 equiv) in DCM (7 mL) were added (*S*)-**3** (109 mg, 0.41 mmol, 1.0 equiv) and DMAP (5 mg, 0.04 mmol, 0.1 equiv). The resulting solution was stirred at RT overnight. The solution was concentrated *in vacuo*. The residue was diluted with EtOAc (10 mL) and washed thoroughly with a sat. aq. $NaHCO_3$ solution (5 mL) and brine (5 mL), dried over $MgSO_4$, filtered and concentrated *in vacuo*. Purified *via* the Biotage SP4 (silica-packed SNAP column 10 g; 20–50 % EtOAc/hexanes) to give **(*S*)-HZ00** as a white solid (110 g, 80% over 2 steps). mp 116-118 °C; $[\alpha]_D^{20}$ = –71.8 (*c* 0.1, $CHCl_3$); Chiral HPLC analysis Chiralpak AD-H (5 % IPA/hexane, 1 mL min^{-1}, 254 nm, 30 °C) t_R (major) 35.5 min, t_R (minor) 31.5 min, >99 % ee; IR (KBr) ν_{max}: 3443 (NH), 3288, 2922, 1658 (C=O), 1530; ^{1}H NMR (500 MHz, $CDCl_3$) δ 8.53 (ddd, J = 4.8, 1.7, 1.1 Hz, 1H, C6'-H̲), 8.26 (dt, J = 7.7, 1.1 Hz, 1H, C3'-H̲), 8.24–8.22 (m, 1H, NH̲), 7.87 (td, J = 7.7, 1.7 Hz, 1H, C4'-H̲), 7.69 (s, 1H, C3-H̲), 7.48 (td, J = 7.7, 1.7 Hz, 1H, ArH̲), 7.46–7.36 (m, 2H, ArH̲, C5'-H̲), 7.31–7.18 (m, 2H, 2 × ArH̲), 5.41–5.25 (m, 1H, C4-H̲), 2.68–2.43 (m, 2H, C7-H̲$_2$), 2.25–2.06 (m, 1H, C5-H̲), 2.02–1.83 (m, 3H, C5-H̲, C6-H̲$_2$); ^{13}C NMR (126 MHz, $CDCl_3$) δ 163.8 (CO), 156.4 (d, J = 251.6 Hz, C), 150.1 (C2'), 148.2 (C6'), 141.9 (C7a), 139.5 (C3), 137.5 (C4'), 130.1 (d, J = 7.8 Hz, CH), 128.7 (CH), 127.5 (d, J = 11.8 Hz, C), 126.3 (C5'), 124.9 (d, J = 3.8 Hz, CH), 122.4 (C3'), 118.1 (C3a), 116.8 (d, J = 20.0 Hz, CH), 42.5 (C4), 30.3 (C5), 21.7 (C7), 20.3 (C6); *m/z* (ES^+) 358.89 ($[M + Na]^+$, 100 %); HRMS (ES^+) Calcd for $C_{19}H_{18}N_4ONa$ $[M + Na]^+$: 359.1284, found 359.1276.

Chiral Separations were performed using a SFC Waters Investigator system with Waters 2998 PDA detector. The column temperature was set to 45 °C.

Synthesis of (*R*)-N-(1-(2-fluorophenyl)-4,5,6,7-tetrahydro-1*H*-indazol-4-yl)-4,5,6,7-tetrahydrobenzo[c]isoxazole-3-carboxamide, (*R*)-HZ05

The reaction scheme for this synthesis has been summarized in supplementary figure 13c. Propylphosphonic anhydride solution (1.15 mL, 2.0 mmol) was added to a slurry of (*R*)-1-(2-fluorophenyl)-4,5,6,7-tetrahydro-1H-indazol-4-amine hydrochloride (S15) (268 mg, 1.00 mmol), 4,5,6,7-tetrahydrobenzo[c]isoxazole-3-carboxylic acid (167 mg, 1.00 mmol) and triethylamine (0.70 mL, 5.0 mmol) in THF. The mixture was stirred at RT for 1 h. The reaction mixture was chromatographed through a short silica column eluted with Heptanes:EtOAc (1:1). The solvents were evaporated and the residue was recrystallized from methanol to give the title compound as a solid (300 mg, 52%). ^{1}H NMR (400 MHz, METHANOL-d_4) d ppm 7.64 (s, 1 H), 7.54 (m, 1H), 7.47 (m, 1H), 7.36 (s, 2H), 5.25 (m, 1H), 2.85 (d, J=1.26 Hz, 2 H), 2.77 (s, 2 H), 2.65-2.47 (m, 2H), 2.15-1.99 (m, 2H), 1.93-1.73 (m, 6H). All ^{1}H NMR data have been detailed in supplementary figure 19b. ^{13}C NMR (101 MHz, METHANOL-d_4) d ppm 163.52 (s) 159.08 (s) 157.20 (s) 158.24 (d, J=251.28 Hz) 143.74 (s) 140.18 (s) 132.23 (d, J=7.41 Hz) 130.09 (s) 128.21 (d, J=12.13 Hz) 126.35 (d, J=4.04 Hz) 120.69 (s) 119.33 (s) 117.92 (d, J=19.54 Hz) 43.88 (s) 30.88 (s) 23.25 (s) 23.06 (s) 22.59 (s) 22.42 (d, J=3.37 Hz) 21.53 (s, 2 C). All ^{13}C NMR data have been detailed supplementary figure 19c. *m/z* (ES^+) 381 $[M+H]^+$; Analysis by SFC, Chiral Cellulose SB column, 4.6 × 150 mm, eluent 20 % methanol in supercritical carbon dioxide, 5 mL min^{-1}, RT 1.70 min (S-isomer RT 3,75 min).

Alternatively N-[1-(2-fluorophenyl)-4,5,6,7-tetrahydro-1H-indazol-4-yl]-4,5,6,7-tetrahydro-2,1-benzoxazole-3-carboxamide (1 g) was dissolved in methanol (12 mL) and DCM (3 mL) and purified

by SFC, injecting 150 µL per run in stacked injections on a Chiral Cellulose column SC (YMC), 250 × 10 mm eluting with CO_2, 20 % methanol, 15 mL min^{-1}. The solvents were evaporated and the solids dried under vacuum to give a solid (365 mg).

In vitro pharmacokinetic analysis

Chemical Stability

Compound HZ00 racemic mix (1 µM from 10 mM DMSO stock) was incubated in closed glass-vials at 37 °C in PBS at pH 7.4 and in PBS:Acetonitrile (1:1) at the same pH for 5 h. Aliquots of the buffer stock solutions were immediately frozen at −80 °C after initiation of the incubation. After the incubation, the samples were analyzed by LC-MS/MS and compared to frozen control samples.

Plasma protein binding

Equilibrium dialysis of drug in plasma against an isotonic buffer was used to determine plasma protein binding of HZ00 racemic mix. Rapid Equilibrium Dialysis (RED) device inserts which allow for short dialysis times (2−4 h) in a 96-well format were used. Pooled human plasma was provided by Uppsala Academic Hospital and was collected from two male and two female donors (non-smoking). In brief, 0.2 mL of the plasma test solution (10 µM final compound concentration) was transferred to the membrane tube in the RED insert. 0.35 mL isotonic phosphate buffer pH 7.4 was added to the other side of the membrane. The 96-well base plate was then sealed with an adhesive plastic film to prevent evaporation. The sample was incubated with rotation (≈900 rpm) on a Kisker rotational incubator at 37 °C for 4 h. A stability test of the test solution is also prepared. For this, 100 µL of the plasma test solution were incubated at 37 °C for 4 h. The plasma test solution was frozen directly after the administration. After incubation, the contents of each plasma and buffer compartment were removed and immediately frozen until analysis. Prior to LC-MS/MS analysis the samples were mixed with equal volumes of control buffer or plasma as appropriate. Plasma proteins were then precipitated by the addition of methanol (1:4) containing warfarin as analytical internal standard. The plate was then sealed, centrifuged and the supernatant analyzed by mass spectrometry (LC-MS/MS).

Calculations: The fraction drug bound was determined using the relationship in equation 1, where plasma concentration and free concentration are the mass spectrometric responses obtained from analysis of the plasma and buffer compartments, respectively. The plasma stability is calculated according to the expression in equation 2. The degree of protein binding, fraction unbound (*fu*) is in general classified according to: *fu* > 50 % low binding, 50 % < *fu* < 10 % moderate binding, 10 % < *fu* < 1 % high binding and *fu* < 1 % very high binding.

The *fu* is calculated using:

$$fu = \frac{C_{\text{unbound}}}{C_{\text{total}}} = \frac{C_{\text{buffer dialysate}}}{C_{\text{plasma dialysate}}} \qquad \textit{equation 1}$$

The stability test is calculated using:

$$\frac{C_{\text{stability incubation}}}{C_{\text{test solution}}} \cdot 100\ \% \qquad \textit{equation 2}$$

>80 % is considered stable.

Metabolic stability

The microsomal metabolic stability assay was measured using pooled human liver microsomes with supplemented cofactor (NADPH). 1 µM HZ00 racemic mix and 0.5 mg mL^{-1} microsomes were diluted in 0.1 M phosphate buffer pH 7.4. The reaction was initiated with addition of 1 mM NADPH. The incubation times were 0, 5, 15, 40 min (n=2) and the reaction was quenched at each time point by addition of acetonitrile containing warfarin as analytical internal standard. The plate was then sealed, centrifuged and frozen at –20 °C until LC-MS/MS analysis.

Calculations: The natural logarithm of the analytical peak area ratio (relative to the 0 min sample which is considered as 100 %) was plotted against time and analyzed by linear regression. The slope of the fitted line is the first-order rate constant of the enzymatic reaction, (assuming the incubation concentration $[S] < 10\ \%\ K_M$ and that Michaelis-Menten kinetics apply). The calculations of *in vitro* half-life and *in vitro* intrinsic clearance (*in vitro* Cl_{int}) are summarized as supplementary equations 3-6[3-4]. The Cl_{int} can be used to rank order compounds but also gives an estimate on the risk of high first-pass metabolism of the liver.

A general classification is, $Cl_{int} < 47$ (µL min^{-1} mg^{-1}) no risk for high first pass metabolism *in vivo*, $47 < Cl_{int} < 92$ moderate risk and $Cl_{int} > 92$ high risk[5].

$$\frac{V_{max}\cdot[S]}{K_M+[S]} \text{ when } [S] \ll K_M \text{ the rate simplifies to } v=\frac{V_{max}\cdot[S]}{K_M} \qquad \textit{equation 3}$$

$$\text{The slope, } k=\frac{V_{max}}{K_M} \qquad \textit{equation 4}$$

$$\textit{in vitro } t_{1/2}(\text{min})=\ln\frac{2}{k} \qquad \textit{equation 5}$$

$$\textit{in vitro } Cl_{int}=\frac{V_{max}}{K_M\left(\frac{\mu L_{incubation}}{mg_{incubation}}\right)\left(\frac{\mu L}{\text{min mg}}\right)} \qquad \textit{equation 6}$$

Caco-2 permeability

The Caco-2 study was performed in accordance with published protocols[4]. Caco-2 cell monolayers (passage 94-105) were grown on permeable filter support and used for transport study on day 21 after seeding. A 1 µM solution of compound was prepared and pre-warmed to 37 °C. The Caco-2 filters were washed with pre-warmed HBSS prior to the experiment. The experiment was started by applying the donor solution on the apical side. The transport experiments were carried out at pH 6.5 in the apical chamber, and pH 7.4 in the basolateral chamber. The experiments were performed at 37 °C and with a stirring rate of 500 rpm. The receiver compartment was sampled at 15, 30 and 60 min, and at 60 min also a final sample from the donor chamber was taken in order to calculate the mass balance of the compound.

The filter inserts were then washed with prewarmed HBSS and the membrane integrity was checked by transepithelial electrical resistance (TEER) and mannitol permeability.

LC-MS/MS analysis

A Waters XEVO TQ triple quadrupole mass spectrometer (electrospray ionization, ESI) coupled to a

Waters Acquity UPLC (Waters Corp.) was used for all LC/MS and LC/MS/MS experiments. For chromatographic separation a general gradient was used (5 % mobile phase B to 90 % over 2 min total run) on a C18 BEH 1.7 µm column 2 × 50 mm (Waters Corp.). Mobile phase A consisted of 5 % acetonitrile 0.1 % formic acid and mobile phase B 100 % acetonitrile 0.1 % formic acid. The flow rate was 0.5 mL min^{-1} and 3 µL of the sample was injected. A standard curve between 3−1000 nM was prepared for each compound. The method sensitivity had an estimated limit of quantitation of < 1 nM.

Cocrystallization, data collection and structure determination of DHODH with inhibitor HZ05

Co-crystals were prepared similar to previously described[6]. In short 18 mg mL^{-1} DHODH (dissolved in 10 mM N,N-dimethylundecylamine N-oxide (C11DAO) (Fluka), 400 mM NaCl, 1 mM EDTA, 100 mM HEPES (pH 7), and 30 % glycerol) was mixed in a 1:1 ratio with 2 M NH_4SO_4, 0.1 M Na Acetate pH 4.8, 30 mM N,N-dimethyldodecylamine N-oxide (DDAO), 15 mM C11DAO, 2 mM L-dihydroorotic acid, 1 mM HZ05 and 6 % DMSO. The crystals did grow from hanging drops at 20°C in a VDX plate; 2.5 µL protein solution mixed with 2.5 µL reservoir (30 % (v/v) glycerol, 0.1 M Na acetate pH 4.8 and 2 M NH_4SO_4). A crystal was flashfrozen in liquid nitrogen and data was collected to 1.7 Å at Diamond Light Source beamline i02. The structure was determined using Molecular Replacement and refined in Refmac5[7]. The coordinates and the structure factors have been deposited in the protein data bank (pdb code: 6ET4) for more details, see Supplementary Table 9.

***In vivo* Pharmacokinetic Parameter Determination**

(*R*)-HZ05 was formulated for subcutaneous and oral administration at 5 mg mL^{-1} in 20 % hydroxypropyl-β-cyclodextrin in PBS and formulated at 5 mg mL^{-1} diluted in physiological NaCl. Female C57B1/6N mice of 6-weeks of age and of a similar body weight were dosed with either 50 mg kg^{-1} subcutaneous or oral formulation in a volume of 10 mL kg^{-1} or dosed with 10 mg kg^{-1} IV and sacrificed by isoflurane administration followed by cervical dislocation after 24 h. Blood was collected in EDTA tubes from three mice per time point and stored on ice with plasma separated within 30 min by centrifugation at 2000 *g* and stored at –80 °C until analysis at 0.08, 0.25, 0.5, 1, 2, 3, 4, 6 and 24 h post administration. 10 mL internal standard (9 nM warfarin) was added to each plasma sample and each sample was shaken at RT for 10 min followed by centrifugation at 2000 *g* for 30 min at 4 °C. 10 mL of sample was injected into an Acquity UPLC coupled to a triple quadrupole mass spectrometer (Waters) operated in MRM mode with positive electrospray ionization. MRM transition 381.09 > 214.99 (28 V for CV and 22 V for CE) was used as a quantifier ion while 381.09 > 199.94 (28 V for CV and 40 V for CE) as a qualifier ion. Transition for internal standard was 309.16 > 163.005 (22 V for CV and 14 V for CE). Ion source conditions were: capillary voltage, 2000 V; desolvation temperature, 500 °C; desolvation gas flow rate, 1000 L h^{-1}. Separation was conducted using a BEH C18 column (2.1 × 50 mm, 1.7 µm, Waters) using a linear gradient: 0 – 0.5 min, 5 % B; 1.2–16 min, 100 % B, 1.61–2.00 min, 5 % B. Mobile phase A was 5 % acetonitrile with 0.1 % formic acid and B was 99.9 % acetonitrile with 0.1 % formic acid. All animal experimentation was carried out by Sweden Contract In vivo Design AB in compliance with 2010/63/EU directive and approved by Stockholms Norra Djurförsöksetiska Nämnd (laboratory animal ethical committee Stockholm) with the approval number N61/15. Any single plasma concentration value deviated by >10 times from the other two values was excluded from the pharmacokinetic evaluation, but was included in the figure.

(R)-HZ00 was formulated for a single intraperitoneal administration at 15 mg mL^{-1} in 10 % DMSO, 40 % PEG-400 in 50% Milli Q H_2O. Nutlin-3 was formulated for a single oral administration at 10 mg mL^{-1} solution in 0.5% tween 80, 2 % w/v HPMC and 97.5 % Milli Q H_2O. Healthy female NOD-SCID mice aged between 8-12 weeks old weighing between 20-35 g were used for the study. 27 mice were divided between three equal groups in a non-blinded manner. One group was dosed single dose of *(R)*-HZ00 at 150 mg kg^{-1}, the second a single dose 100 mg kg^{-1} nutlin-3 and the third group co-administered one single dose of 150 mg kg^{-1} *(R)*-HZ00 and one single dose of 100 mg kg^{-1} nutlin-3. Dosing was 10 mL kg^{-1} for both routes of administration. Blood samples of approximately 60 µL were

collected from the retro-orbital plexus of three mice per group per time point at the following times: pre-dose, 0.08, 0.25, 0.5, 1, 2, 4, 8, and 24 h. Samples were collected into tubes with 20 % potassium-EDTA solution. Plasma was separated at 4000 rpm for 10 minutes at 4 ˚C and stored at -70 ˚C until analysis. 25 μL of study sample or spiked plasma calibration standards were added to individual tubes followed by 100 μL of Glipizide at 500 ng mL^{-1} as an internal standard. Samples were centrifuged at 4000 rpm at 4 ˚C for 10 minutes. 100 μL of cleared supernatant was transferred to 96-well plates and analyzed using a Waters ACQUITY UPLC connected to an AB Sciex API 4000 QTRAP in MRM mode with positive electrospray ionization with an ion spray voltage of 5500 V. The mobile phase was A: 0.1 % formic acid in acetonitrile and B: 0.1 % formic acid in water through a phenomenex Kinetex EVO C18 (5 μm, 100 × 4.6 mm) kept at 45 ˚C. LC conditions are as follows, flow rate 1 mL min^{-1}. A stepped gradient was used as follows: 20 % A at 0 min to 95 % A at 0.8 min held until 2.2 min, to 20 % A at 2.6 min and held until 3 min. MRM transitions are as follows: HZ00 337.2 > 199.3 with 60 V declustering potential, 54 V CE and 8 V CXP with a dwell time of 40 ms; nutlin-3 581.2 > 438.9 with 96 V declustering potential, 42 V CE and 15 V CXP with a dwell time of 40 ms; glipizide 446.3 > 347.0 with 40 V declustering potential, 22 V CE and 12 V CXP with a dwell time of 40 ms. All animal studies were carried out by SAI Life India in accordance with the guidelines provided by the Committee for the Purpose of Control and Supervision of Experiments on Animals (CPCSEA) as published in The Gazette of India, December 15, 1998. Prior approval of the Institutional Animal Ethics Committee (IAEC) was obtained before initiation of the study. The IAEC number for this study is FB-16-048.

(R)-HZ05 was formulated for a single subcutaneous administration at 15 mg mL^{-1} in 10% DMSO, 40 % PEG-400 in 50 % Milli Q H_2O. Nutlin-3a was formulated for a single oral administration at 5 mg mL^{-1} solution in 0.5 % tween 80, 2 % w/v HPMC and 97.5 % Milli Q H_2O. Healthy female NOD-SCID mice aged between 8-12 weeks old weighing between 20–35 g were used for the study. 27 mice were divided between three equal groups in a non-blinded manner. One group was dosed single dose of *(R)*-HZ05 at 75 mg kg^{-1}, the second a single dose 50 mg kg^{-1} nutlin-3 and the third group co-administered one single dose of 75 mg kg^{-1} *(R)*-HZ05 and one single dose of 50 mg kg^{-1} nutlin-3. Dosing was 5 mL kg^{-1} for subcutaneous and 10 mg kg^{-1} for oral administration. Blood samples of approximately 60 μL were collected from the retro-orbital plexus of three mice per group per time point at the following times: pre-dose, 0.08, 0.25, 0.5, 1, 2, 4, 8, and 24 h. Samples were collected into tubes with 20 % potassium-EDTA solution. Plasma was separated at 4000 rpm for 10 minutes at 4 ˚C and stored at –70 ˚C until analysis. 25 μL of study sample or spiked plasma calibration standards were added to individual tubes followed by 100 μL of Lansoprazole at 500 ng mL^{-1} as an internal standard. Samples were centrifuged at 4000 rpm at 4 ˚C for 10 minutes. 100 μL of cleared supernatant was transferred to 96-well plates and analyzed using a Waters ACQUITY UPLC connected to an AB Sciex API 4000 QTRAP in MRM mode with positive electrospray ionization with an ion spray voltage of 5500 V. The mobile phase was A: 0.1 % formic acid in acetonitrile and B: 0.1 % formic acid in water through a phenomenex Kinetex EVO C18 (5 μm, 100 × 4.6 mm) kept at 45 ˚C. LC conditions are as follows, flow rate 0.7 mL min^{-1}. A stepped gradient was used as follows: 0 % A at 0 min hold at 0 % A until 0.3 then to 95 % A at 0.4 min held until 1.2 min, to 0 % A at 1.4 min and held until 1.8 min. MRM transitions are as follows: HZ05 381.2 > 214.9 with 39 V declustering potential, 31 V CE and 12 V CXP with a dwell time of 35 ms; nutlin-3 581.2 > 263.2 with 12 V declustering potential, 49 V CE and 15 V CXP with a dwell time of 35 ms; glipizide 370.1 > 252.1 with 46 V declustering potential, 20 V CE and 6 V CXP with a dwell time of 35 ms. All animal studies were carried out by SAI Life India in accordance with the guidelines provided by the Committee for the Purpose of Control and Supervision of Experiments on Animals (CPCSEA) as published in The Gazette of India, December 15, 1998. Prior approval of the Institutional Animal Ethics Committee (IAEC) was obtained before initiation of the study. The IAEC number for this study is FB-16-048.

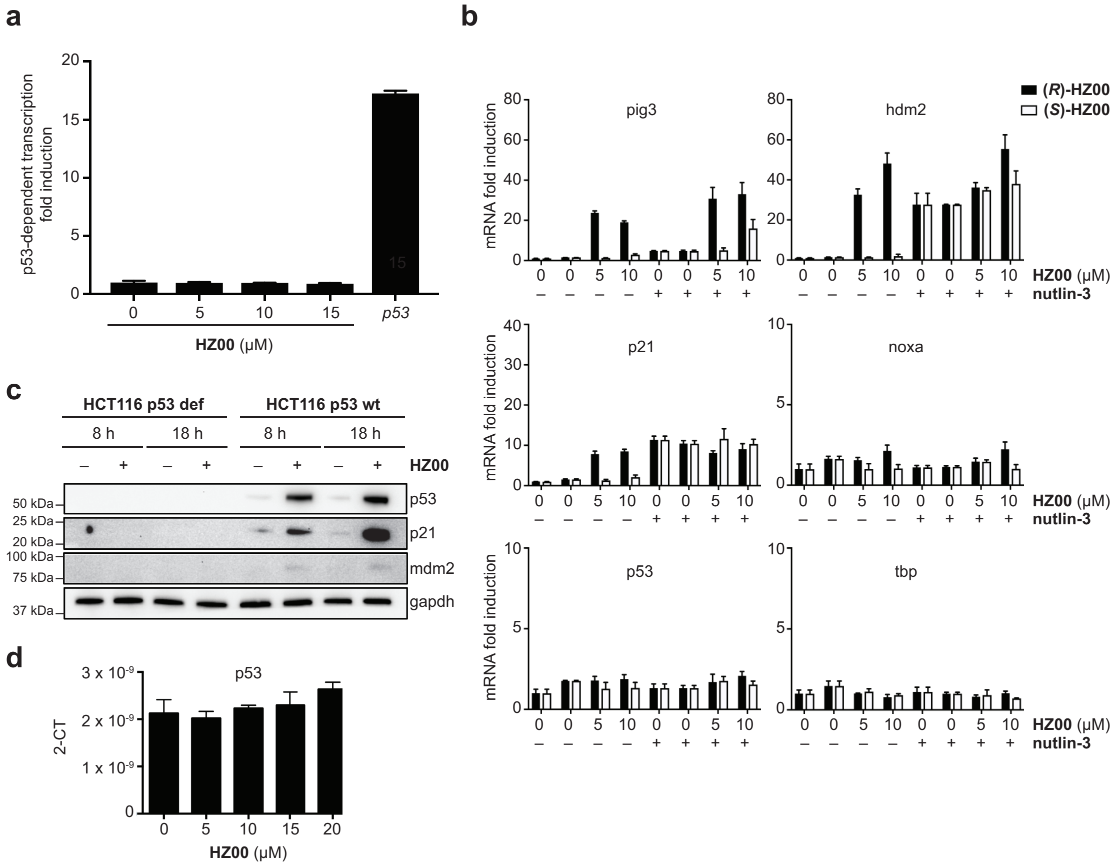

Supplementary Figure 1. HZ compounds increase the levels of p53-induced mRNAs. a) p53-null H1299 cells were transfected with the RGCΔFosLacZ reporter construct and levels of p53-dependent transcription were determined by CPRG assay following a 16 h exposure to HZ00. A vector expressing wild-type p53 was used as a positive control. The average of three technical repeats ± SD is shown. b) ARN8 cells were pretreated with (*R*)-HZ00 or (*S*)-HZ00 for 1 hour prior to treatment with 2 µM nutlin-3 for an additional 18 hours. Levels of p53-induced mRNAs and p53 mRNA were determined by q-RT-PCR. tbp was used as a control. Values correspond to the average of three technical repeats with standard deviation used for error. RNA extraction and q-RT-PCR were performed as previously described - see supplementary information[7]. c) HCT116 p53 wild-type or p53 deficient cells were treated with DMSO or 20 µM HZ00 for the indicated times and analyzed by western blotting. See supplementary information for cell line description[8]. d) ARN8 cells treated for 6 hours with HZ00 at indicated doses. Levels of p53 mRNA were determined by q-RT-PCR.

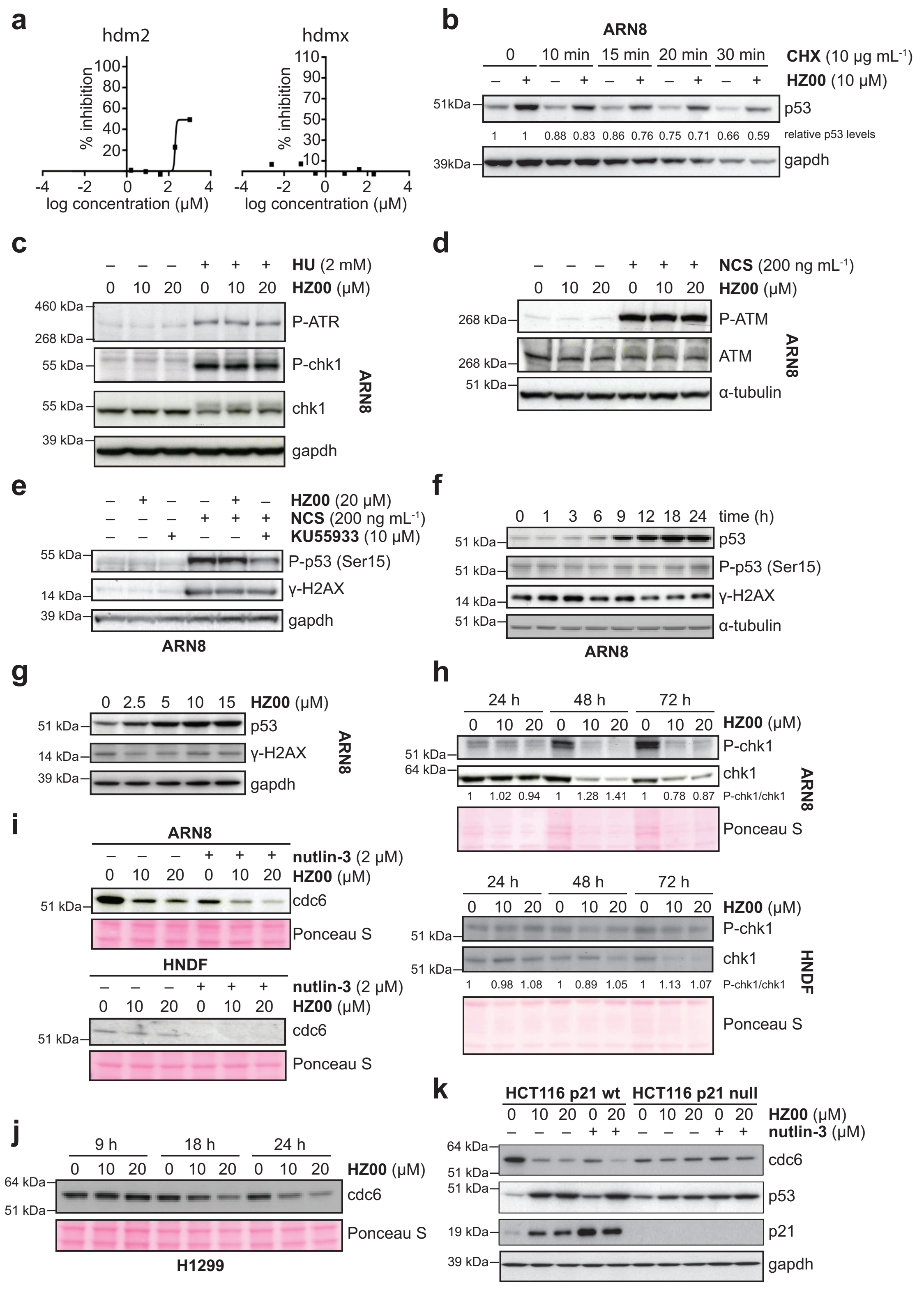

Supplementary Figure 2. HZ00 is mechanistically different to nutlin-3 and does not induce markers of DNA damage. a) Following the incubation of *in vitro* translated hdm2 or hdmx protein with HZ00 or vehicle control, hdm2/hdmx binding to IP3 peptide (which corresponds to residues 17–26 of wild-type p53) was determined by ELISA (See supplementary information[9]). The percentage hdm2/hdmx bound to IP3 relative to the vehicle control was calculated. b) ARN8 cells were treated with HZ00 or vehicle (DMSO) for 7 h, upon which cycloheximide (CHX) (10 μg mL^{-1}) was added for the indicated periods of time.

Protein levels were analyzed by western blotting and relative p53 levels quantified using Adobe Photoshop. gapdh served as loading control. c) ARN8 cells were pretreated with HZ00 for 1 h prior to the addition of hydroxyurea (HU) or vehicle control for a further 3 h and analyzed by western blotting. gapdh was used as loading control. d) ARN8 cells were treated with HZ00 for 1 h before the addition of the DNA damaging agent neocarzinostatin (NCS) (Sigma, #N9162) for a further 2 h. Samples were lysed in 2 × LDS buffer (Invitrogen) and levels of P-ATM and ATM determined. α-tubulin was detected for loading control. e) ARN8 cells were treated with HZ00 and/or the ATM inhibitor KU55933 for 1 h before the addition of NCS for a further 2 h. Samples were lysed as in d) and levels of P-Ser15 p53 and γ-H2AX were determined. gapdh was used as loading control. f–g) ARN8 cells were treated with either 10 μM HZ00 for the indicated times or for 6 h at the indicated concentrations and subjected to western blotting. α-tubulin or gapdh were used as loading controls. h) ARN8 or HNDF cells were treated with HZ00 or vehicle (DMSO) for the indicated times and P-chk1 (Ser 345) and chk1 were detected. Ponceau S staining was used to monitor loading. The ratio of P-chk1 to chk-1 was quantified using Adobe Photoshop. i) ARN8 and HNDF cells were treated with HZ00 for 1 h and then 2 μM nutlin-3 was added for an additional 18 h. cdc6 was detected and Ponceau S staining was used to monitor loading. j) p53-null H1299 cells were treated with HZ00 for 9, 18 or 24 h and levels of cdc6 determined by western blotting. Ponceau S staining was used to monitor loading. k) HCT116 p21 wt cells and HCT116 p21 null cells were treated with HZ00 for 1 hour and then nutlin-3 added for an additional 18 hours (19 hours HZ00 in total) and analyzed by western blotting.

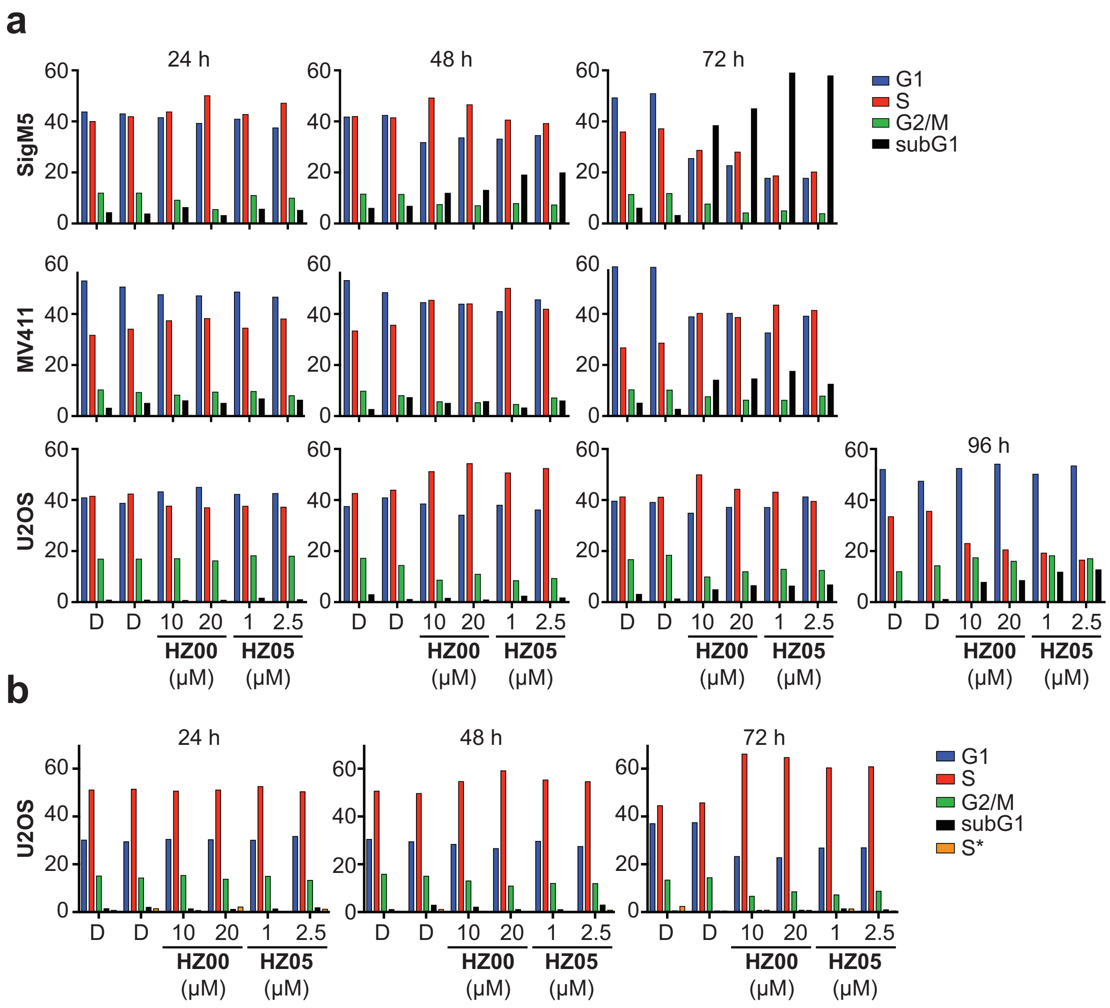

Supplementary Figure 3. HZ00 and HZ05 can lead to the accumulation of p53-wild type cancer cell lines in S-phase. a) p53 wild type cell lines were treated as indicated and analyzed by PI staining and flow cytometry. % of cells in each cell cycle phase was determined by FlowJo using the Watson algorithm. b) U2OS cells were treated as indicated and analyzed by bidimensional flow cytometry. S* indicates cells with a DNA content between 2N and 4N that do not incorporate BrdU. Each cell cycle phase was determined using FlowJo.

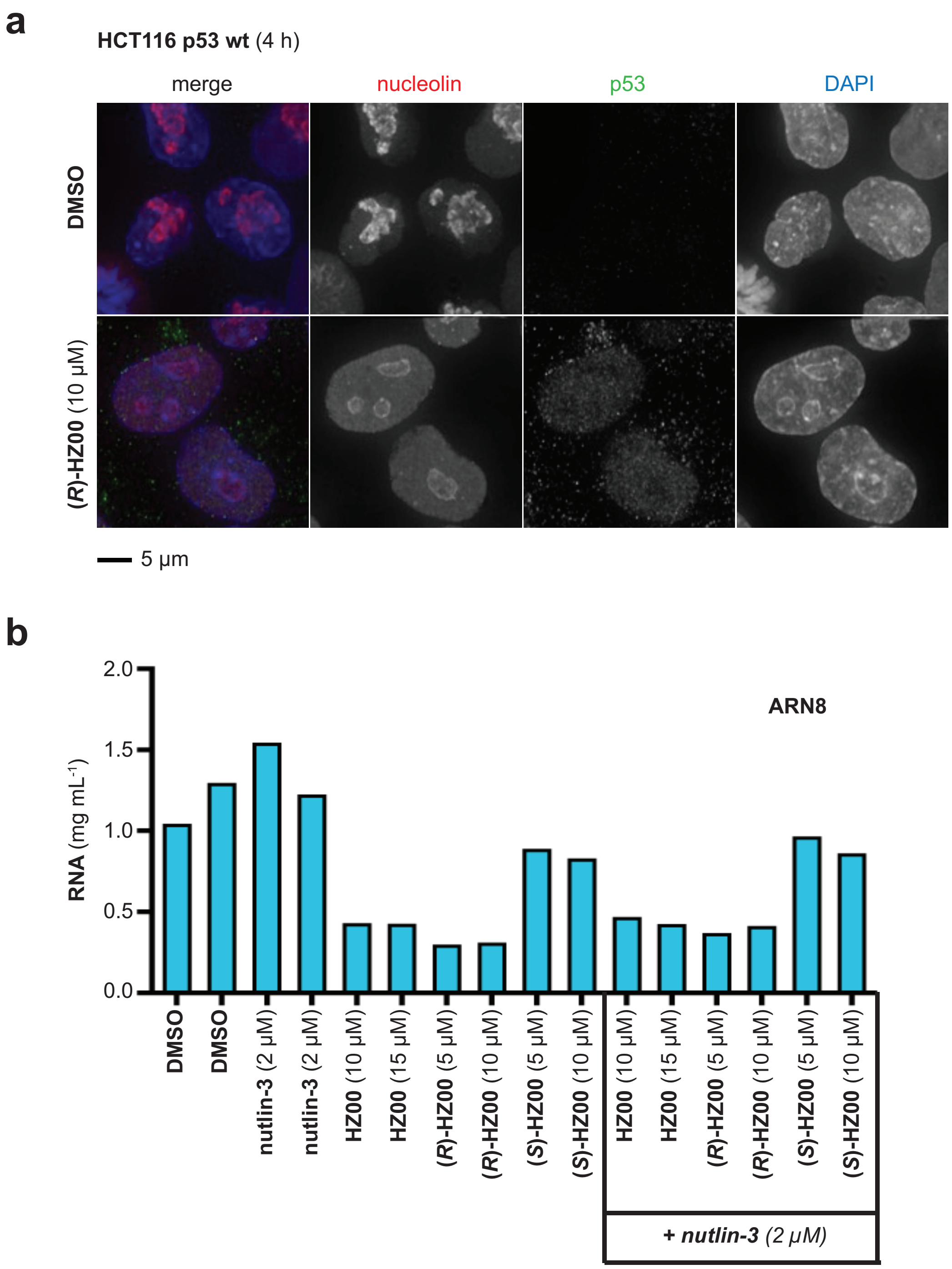

Supplementary Figure 4. (*R*)-HZ00 disrupts nucleoli and reduces total RNA levels. a) HCT116 cells were treated with *(R)*-HZ00 for 4 h. Cells were fixed in 4 % paraformaldehyde made in PBS followed by 10 min at 37 ^{o}C, permeabilization in 0.15 % Triton X-100 for 1-2 min at 37 ^{o}C, and staining with antibodies against nucleolin and p53. Images were taken using Olympus IX-71 microscope controlled by Delta Vision SoftWorx. Image stacks were deconvolved, quick-projected and saved as tiff images to be processed using Adobe PhotoShop. Antibodies to specific antigens are listed in Supplementary Table 8. b) ARN8 cells were pretreated with HZ00, *(R)*-HZ00 or *(S)*-HZ00 for 1 h prior to treatment with 2 µM nutlin-3 for an additional 18 h (i.e., 19 h HZ00, *(R)*-HZ00 or *(S)*-HZ00 in total). Total RNA was extracted and quantified.

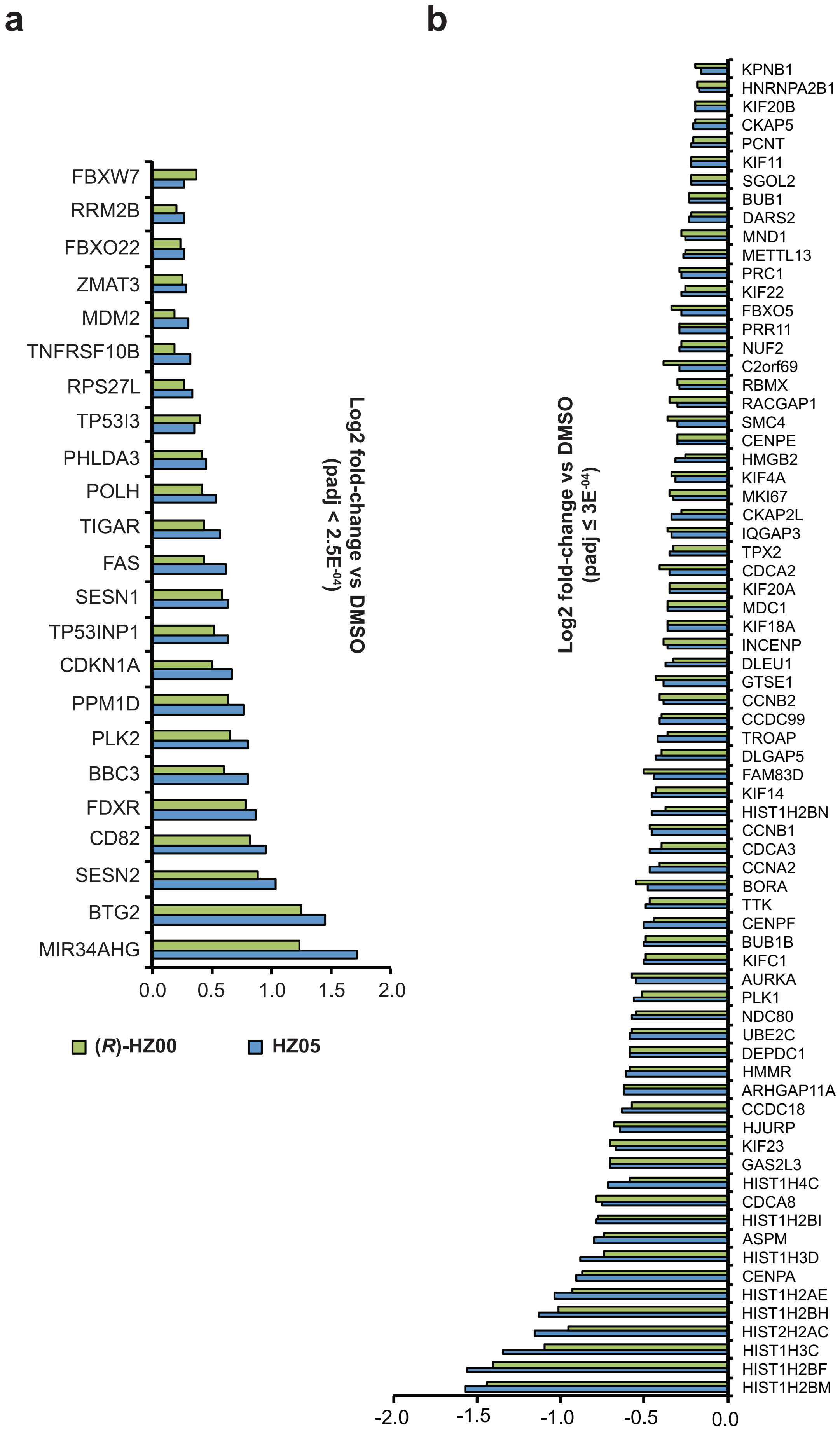

a
b
FBXW7
RRM2B
FBXO22
ZMAT3
MDM2
TNFRSF10B
RPS27L
TP53I3
PHLDA3
POLH
TIGAR
FAS
SESN1
TP53INP1
CDKN1A
PPM1D
PLK2
BBC3
FDXR
CD82
SESN2
BTG2
MIR34AHG
0.0 0.5 1.0 1.5 2.0
Log2 fold-change vs DMSO (padj < 2.5E-04)
(R)-HZ00
HZ05
KPNB1
HNRNPA2B1
KIF20B
CKAP5
PCNT
KIF11
SGOL2
BUB1
DARS2
MND1
METTL13
PRC1
KIF22
FBXO5
PRR11
NUF2
C2orf69
RBMX
RACGAP1
SMC4
CENPE
HMGB2
KIF4A
MKI67
CKAP2L
IQGAP3
TPX2
CDCA2
KIF20A
MDC1
KIF18A
INCENP
DLEU1
GTSE1
CCNB2
CCDC99
TROAP
DLGAP5
FAM83D
KIF14
HIST1H2BN
CCNB1
CDCA3
CCNA2
BORA
TTK
CENPF
BUB1B
KIFC1
AURKA
PLK1
NDC80
UBE2C
DEPDC1
HMMR
ARHGAP11A
CCDC18
HJURP
KIF23
GAS2L3
HIST1H4C
CDCA8
HIST1H2BI
ASPM
HIST1H3D
CENPA
HIST1H2AE
HIST1H2BH
HIST2H2AC
HIST1H3C
HIST1H2BF
HIST1H2BM
-2.0 -1.5 -1.0 -0.5 0.0
Log2 fold-change vs DMSO (padj ≤ 3E-04)

Supplementary Figure 5. List of p53 modulated RNAs altered by HZ compounds. ARN8 cells were treated with HZ05 (5 μM) or (*R*)-HZ00 (20 μM) for 5 h and samples were analysed by RNASeq. Values correspond to the average of three biological repeats. a) Induction of MIR34AHG and of mRNAs for genes suggested as p53 inducible targets (see supplementary methods[10]). b) Repression of mRNAs modulated by the p53-DREAM complex (see supplementary methods[11]).

a

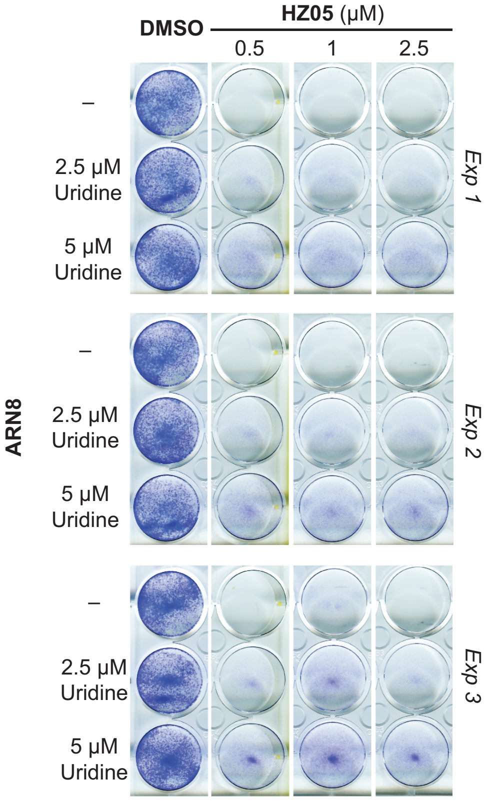

b

		nutlin-3a (µM)				
		**0**	**5**	**10**	**12.5**	**15**
HZ05 (nM)	**0**	0	0.05	0.07	0.10	0.12
	**125**	0.03	0.19	0.39	0.50	0.63
	**250**	0.06	0.27	0.48	0.61	0.69
	**500**	0.08	0.35	0.60	0.58	0.67
	**1000**	0.11	0.3792	0.56	0.62	0.71

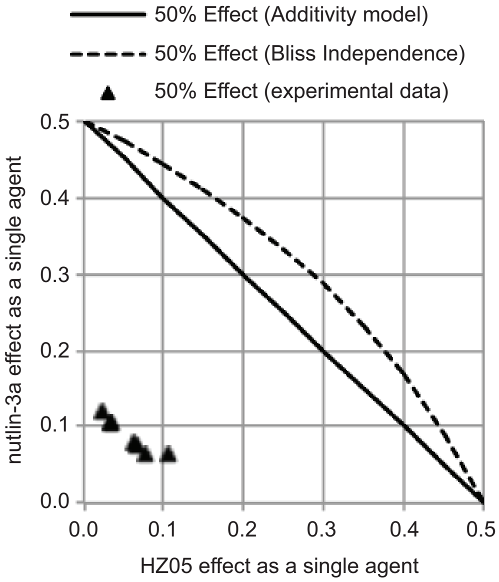

Supplementary Figure 6. Effects of plasma concentrations of uridine on HZ05 activity and synergy of HZ05 with nutlin-3a. a) ARN8 cells were seeded in FBS supplemented medium with a medium change to serum replacement medium 24 hours after seeding. The cells were treated for 72 hours with the indicated dose of HZ05 in the presence of 2.5 or 5 µM uridine. b) ARN8 cells were treated for 72 h with HZ05 and/or nutlin-3a at the indicated doses. After treatment, cell-cycle distribution was analyzed by flow cytometry following staining with PI. The effect of the compounds was quantified by obtaining the percentage of cells in sub-G1. The table shows the DMSO control subtracted effect for each dose combination (d_H, d_N). The curves in the normalized EC_{50} isobologram for the HZ05-nutlin-3a combination indicate single-effect pairs (x, y) = (Eff$[d_H]$/100, Eff$[d_N]$/100) expected to give a 0.5 effect in combination according to the additivity (solid line) and Bliss independence (dashed line) models respectively (see supplementary information[12]). Data points ▲ indicate pairs (x, y) that give 0.5 effect based on linear interpolation of the experimental data shown in the combination matrix above 50 %.

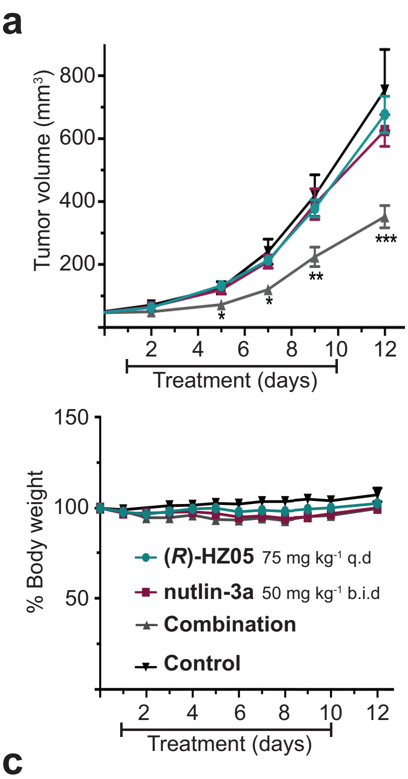

b

Parameter	i.v	p.o	s.c
Dose (mg kg^{-1})	5	50	50
C_{max} (μM)	6.1	7.5	14.9
T_{max} (h)	0.08	0.25	0.5
AUC $_{0\text{-h}}$ (μmol*h L^{-1})	2.4	9.9	30
AUC $_{0\text{-inf}}$ (μmol*h L^{-1})	2.4	9.9	30
λ_z range (h)	1–6	3–24	6–24
t½ (h)	1.1	2.9	2.5
Vd	8.3	-	-
Vss	3.6	-	-
CL	5.4	-	-
BA (%)	-	41	>100

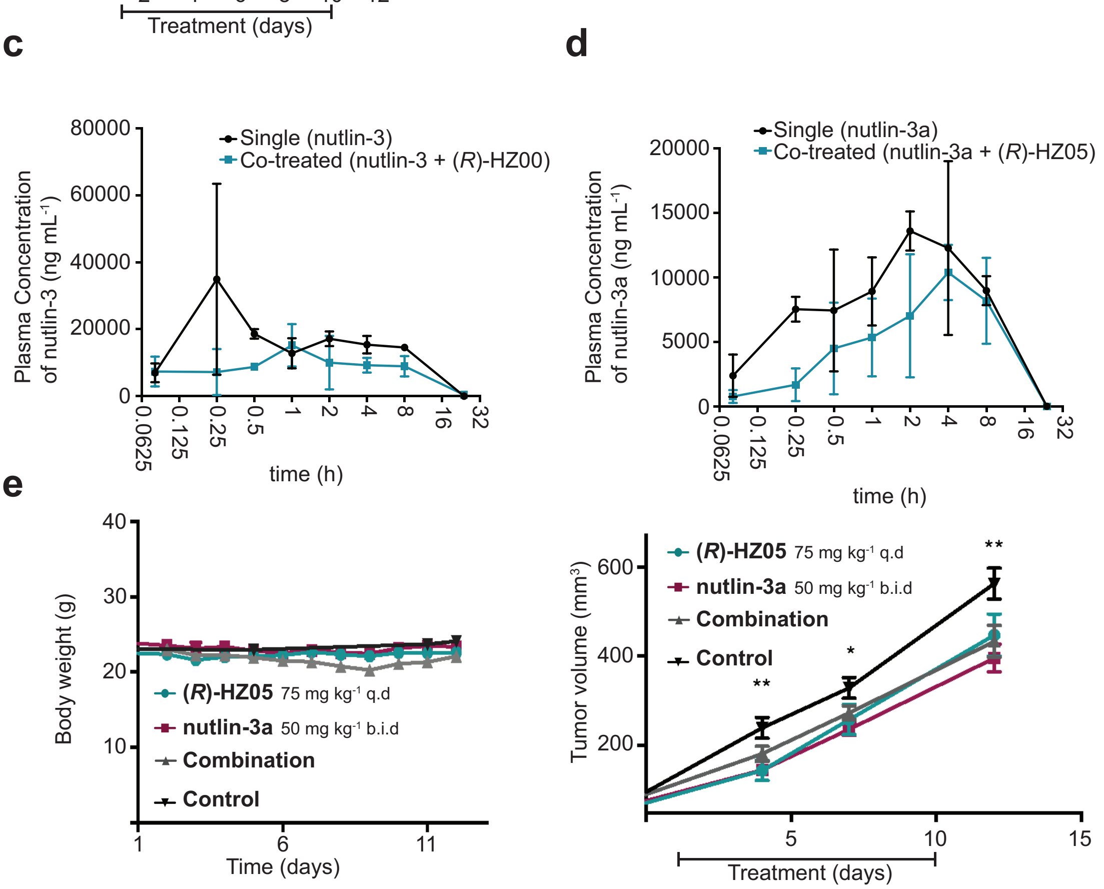

Supplementary Figure 7. Effect of the combination of (*R*)-HZ05 and N3a on ARN8 xenografts and pharmacokinetic properties of (*R*)-HZ05. a) Tumor volume showing inhibition of tumor growth for mice (n = 8 per group) treated for 10 days with a combination of nutlin-3a 50 mg/kg b.i.d. orally and (*R*)-HZ05 75 mg/kg q.d. s.c. in comparison with mice receiving monotherapy or vehicle, in a subcutaneous xenograft model of ARN8. b.i.d. twice a

day; q.d. daily; s.c. subcutaneously. Error bars illustrate ± SEM with significance determined by Multiple Student's *t*-tests. (***$p < 0.001$, **$p < 0.01$ and *$p < 0.05$). Animal weights were monitored as shown in the bottom panel. b) *In vivo* pharmacokinetic parameters of (*R*)-HZ05. c) Pharmacokinetic study examining the effect of co-administration of nutlin-3 and HZ00 on the plasma concentration of nutlin-3 over time. See supplementary information for detailed description of protocol. d) Pharmacokinetic study examining the effect of co-administration of nutlin-3a and (R)-HZ05 on the plasma concentration of nutlin-3a over time. See supplementary information for detailed description of protocol. e) *In vivo* activity of (*R*)-HZ05 alone and in combination with nutlin-3 on H1299 p53-null xenograft tumors ($n \geq 6$). Conditions as in Fig. 2e. In the tumor volume graph, (**) on day 4 indicates a statistical difference of Control to HZ05 and to nutlin-3a; (*) on day 7 indicates a difference between Control and nutlin-3a; and (**) on day 12 indicates a statistical difference between Control, Combination and nutlin-3a. ***$p < 0.001$,**$p < 0.01$ and *$p < 0.05$

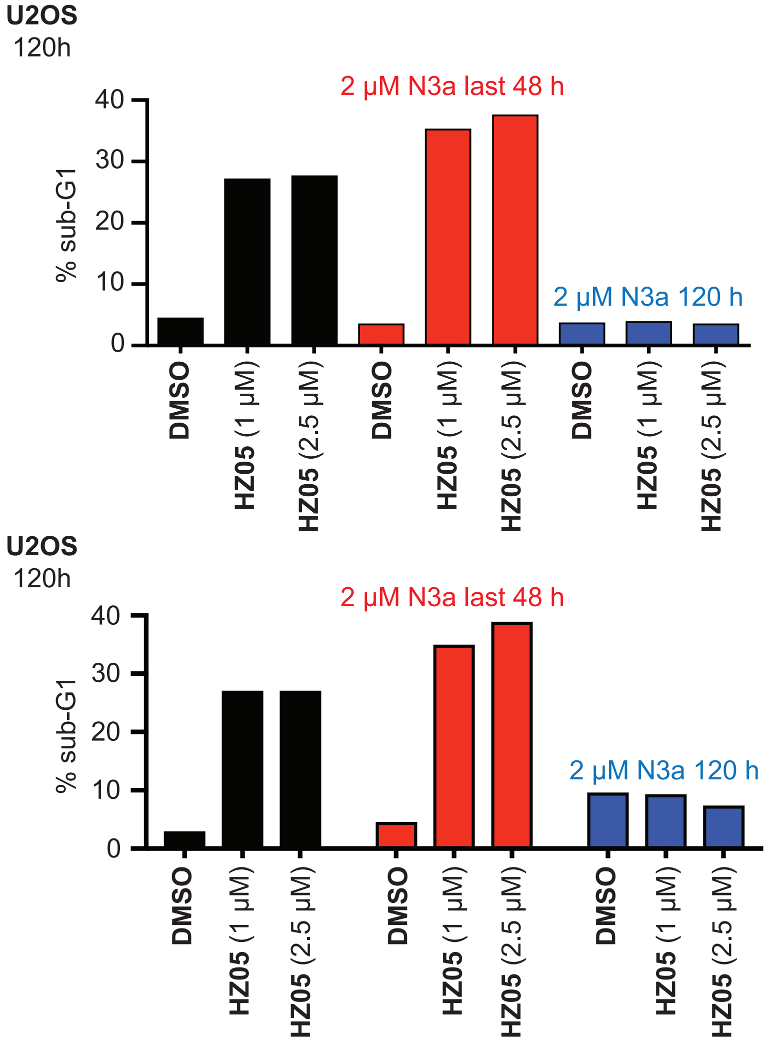

Supplementary Figure 8. Phasing HZ05 and nutlin-3a treatments is necessary to observe nutlin-3a induced cell death in U2OS cultures. U2OS cells were treated with DMSO (vehicle) or HZ05 for 72 h. At this point, an extra dose of DMSO or HZ05 was added and cells were incubated for a further 48 h (black bars). Values shown in red correspond to the same experiment but in the presence of nutlin-3a for the last 48 h. Values shown in blue correspond to the same experiment but where nutlin-3a was added at the same time as HZ05. Two independent experiments are shown.

a

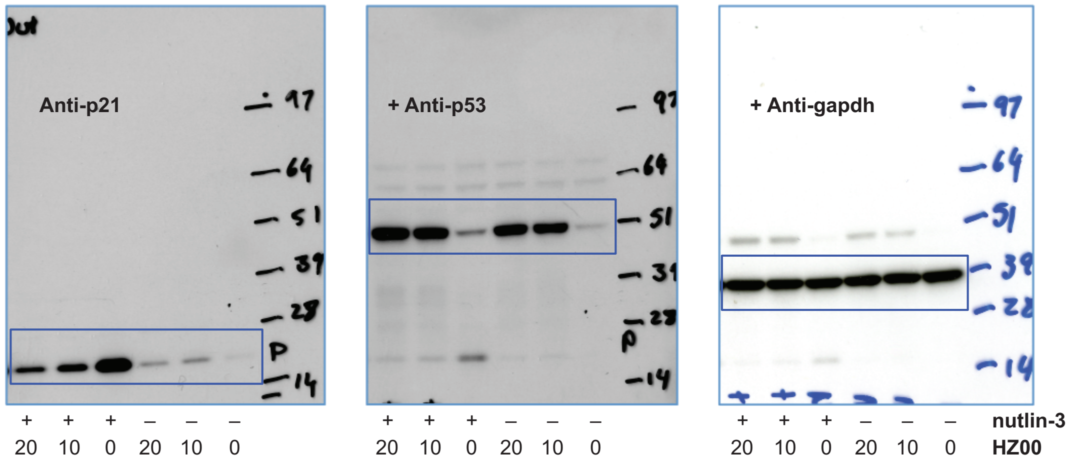

b

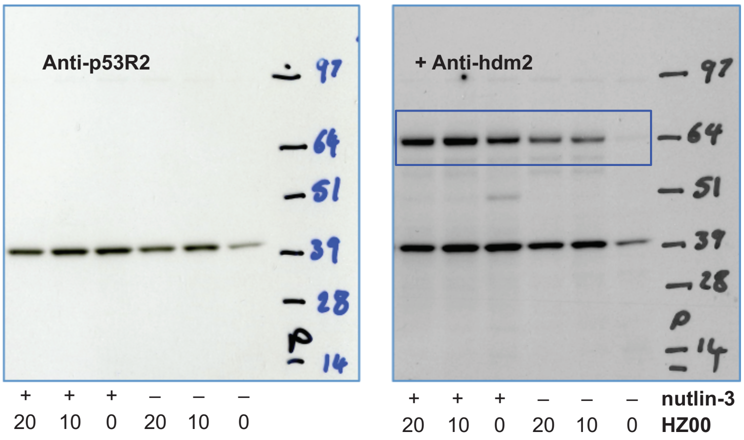

Supplementary Figure 9. Full Blots for Figure 1d. Both blots were run using the same samples. a) Western blot of ARN8 cells incubated with three antibodies sequentially. b) Second membrane of western blotted ARN8 cells incubated with two antibodies sequentially. p53R2 is the product of another p53 target gene and is not included in this manuscript.

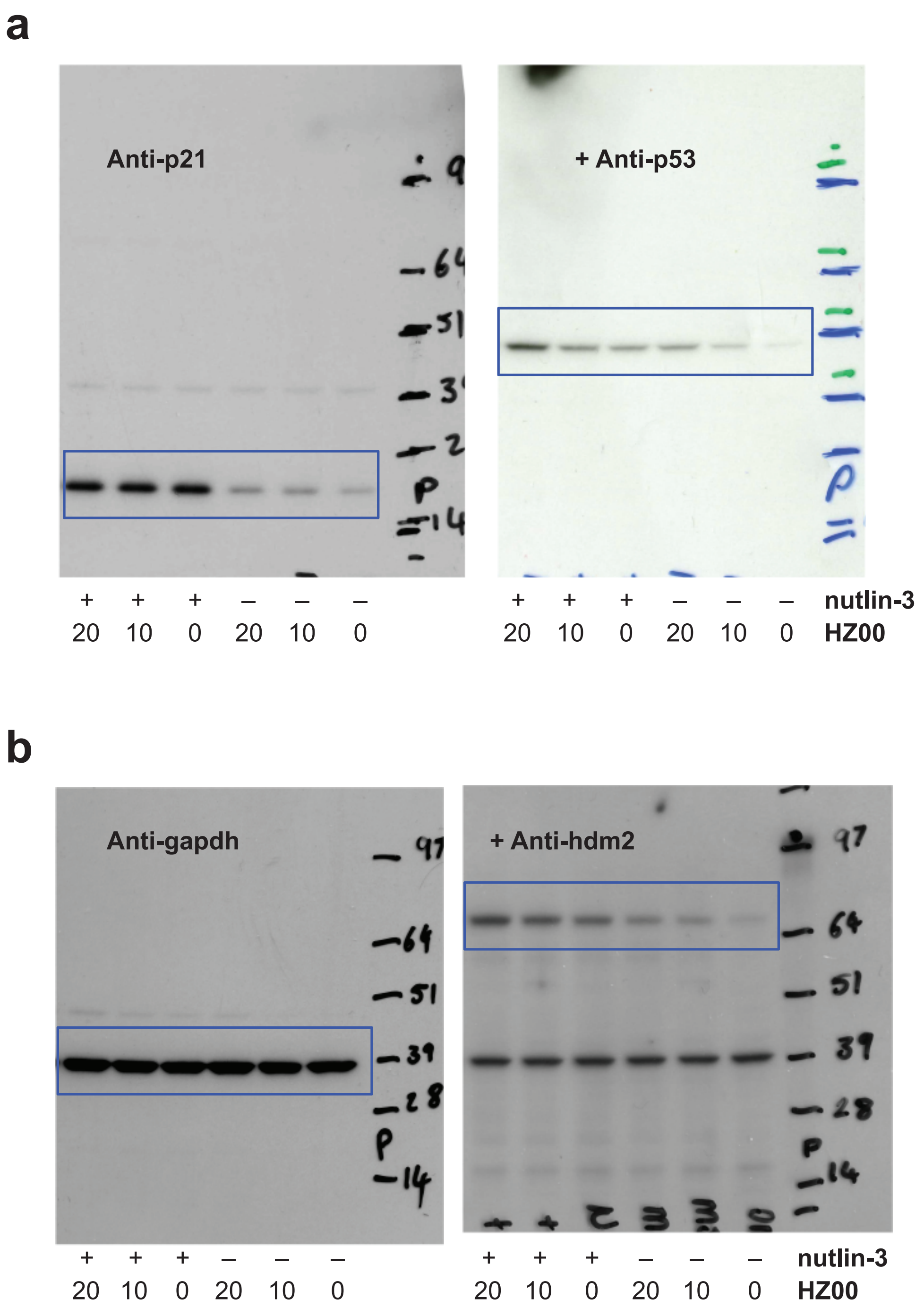

Supplementary Figure 10. Full Blots for Figure 1d. Both blots were run using the same set of samples. a) Western blot of HNDF cells incubated with two antibodies sequentially. b) Second membrane of western blotted HNDF cells incubated with two antibodies sequentially.

a

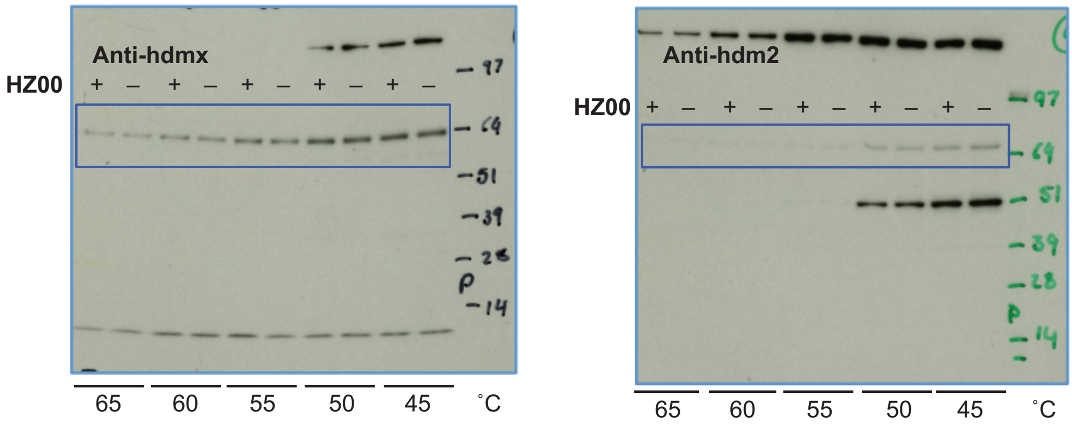

b

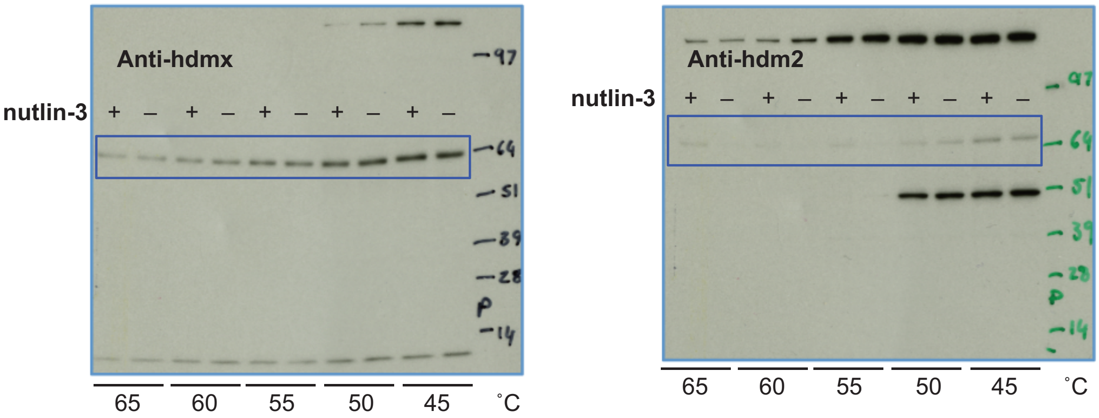

Supplementary Figure 11. Full Blots for Figure 1f. a) Western blot of the cellular thermal shift assay (CETSA) using HZ00. Two separate membranes from the same experiment were run for each antibody. b) Western blot of the CETSA using nutlin-3. Two separate membranes from the same experiment were run for each antibody

a

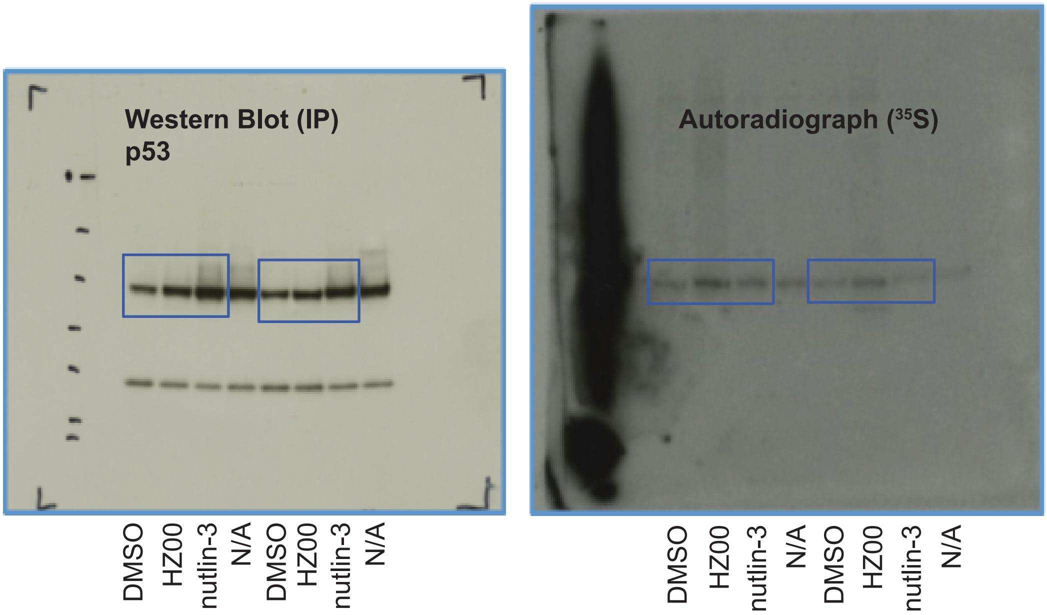

Supplementary Figure 12. Full Blots for Figure 1g. a) Western blot of immunoprecipitation samples with p53 detected and autoradiograph detecting synthesis of p53 following immunoprecipitation of p53.

a

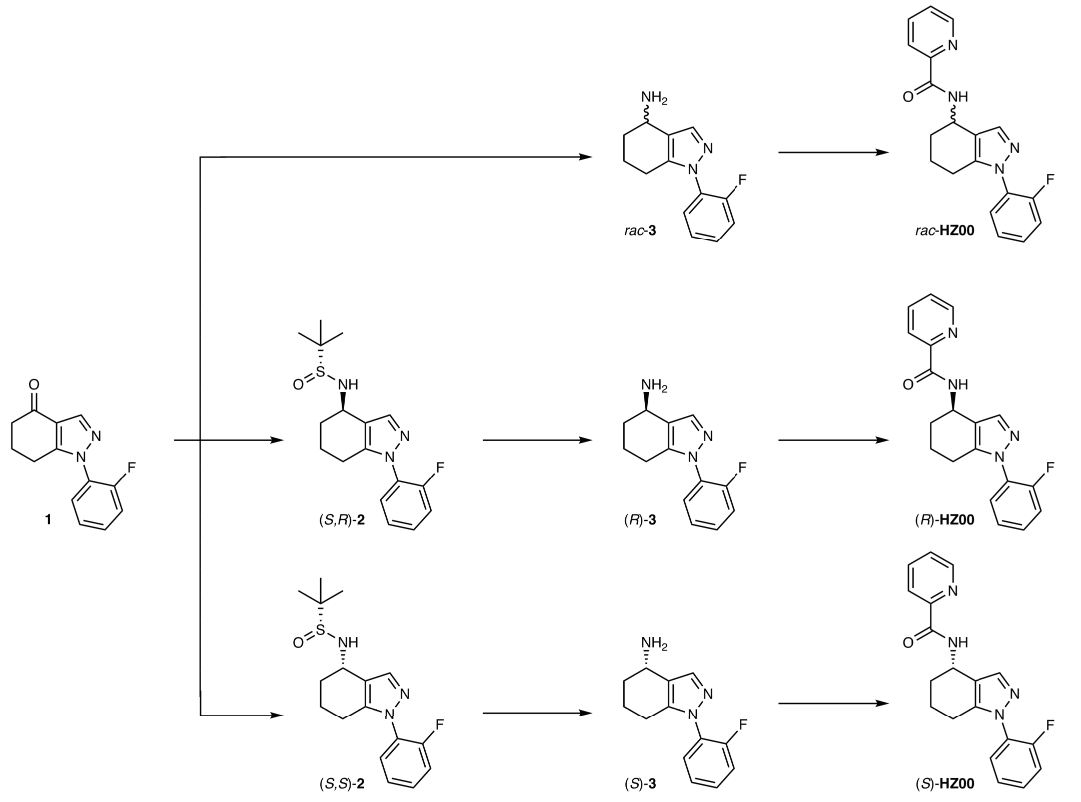

b

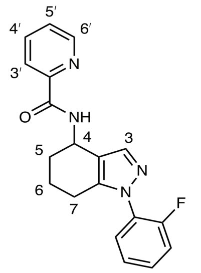

c

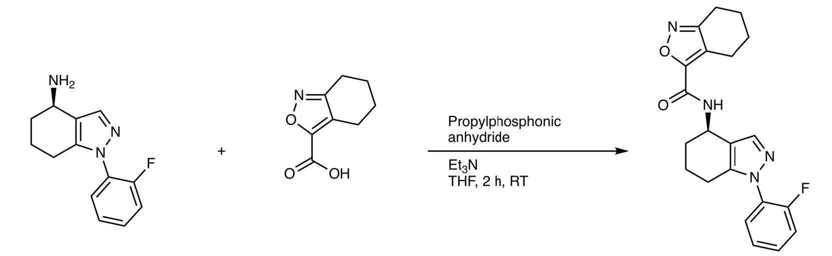

Supplementary Figure 13. Reaction schemes for chemical synthesis of HZ00. a) Reaction scheme for (*rac*)-HZ00, (*S*)-HZ00 and (*R*)-HZ00. b) carbon notation for HZ00. c) synthesis of (*R*)-HZ05

a

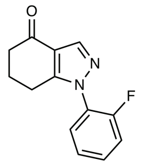

1-(2-Fluorophenyl)-6,7-dihydro-1H-indazol-4(5H)-one (**1**)

b

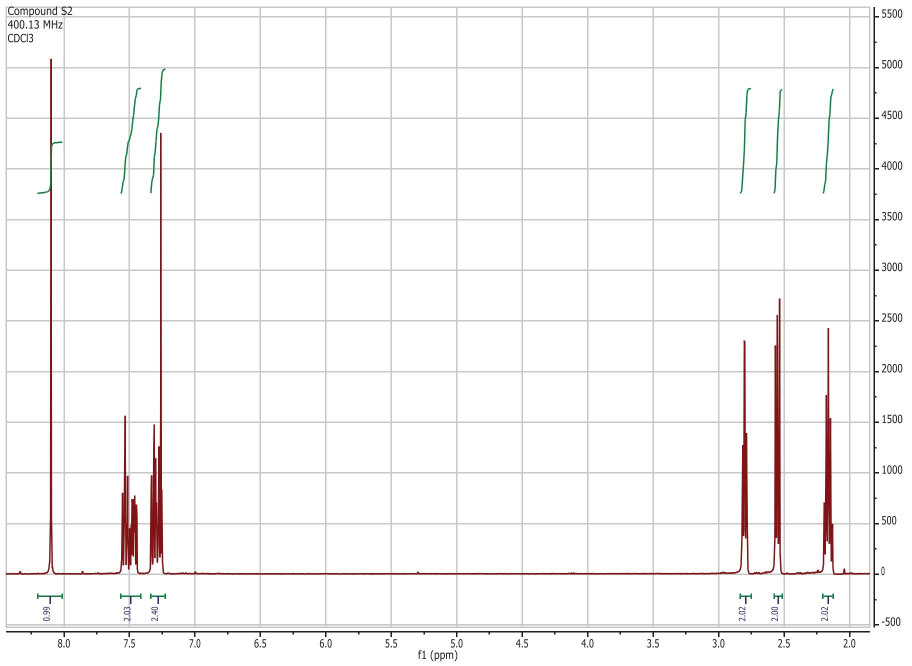

c

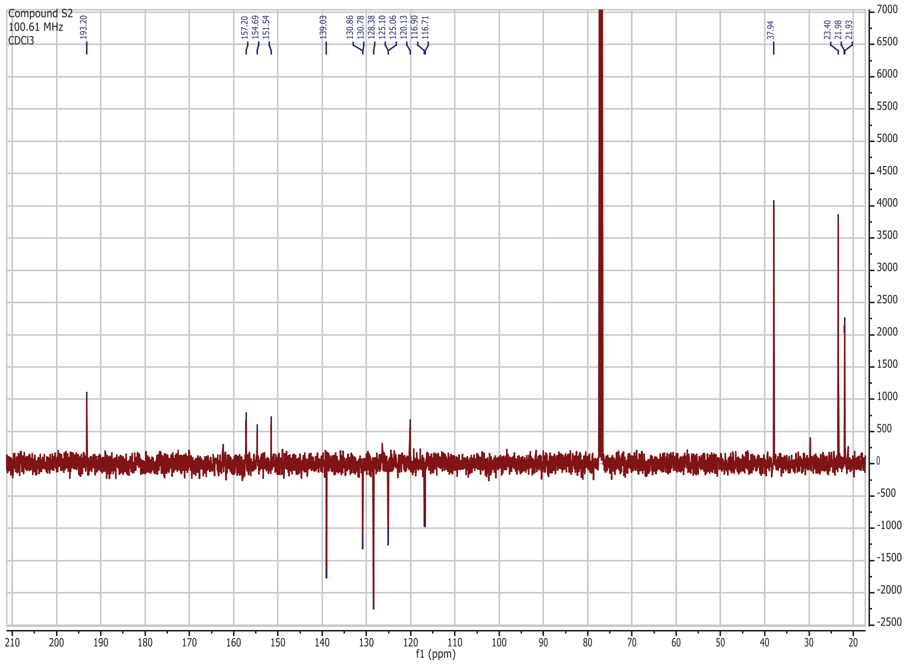

Supplementary Figure 14. Synthesis of (1). a) structure of (1). b) ^{1}H-NMR trace of (1). c) ^{13}C-NMR trace of (1).

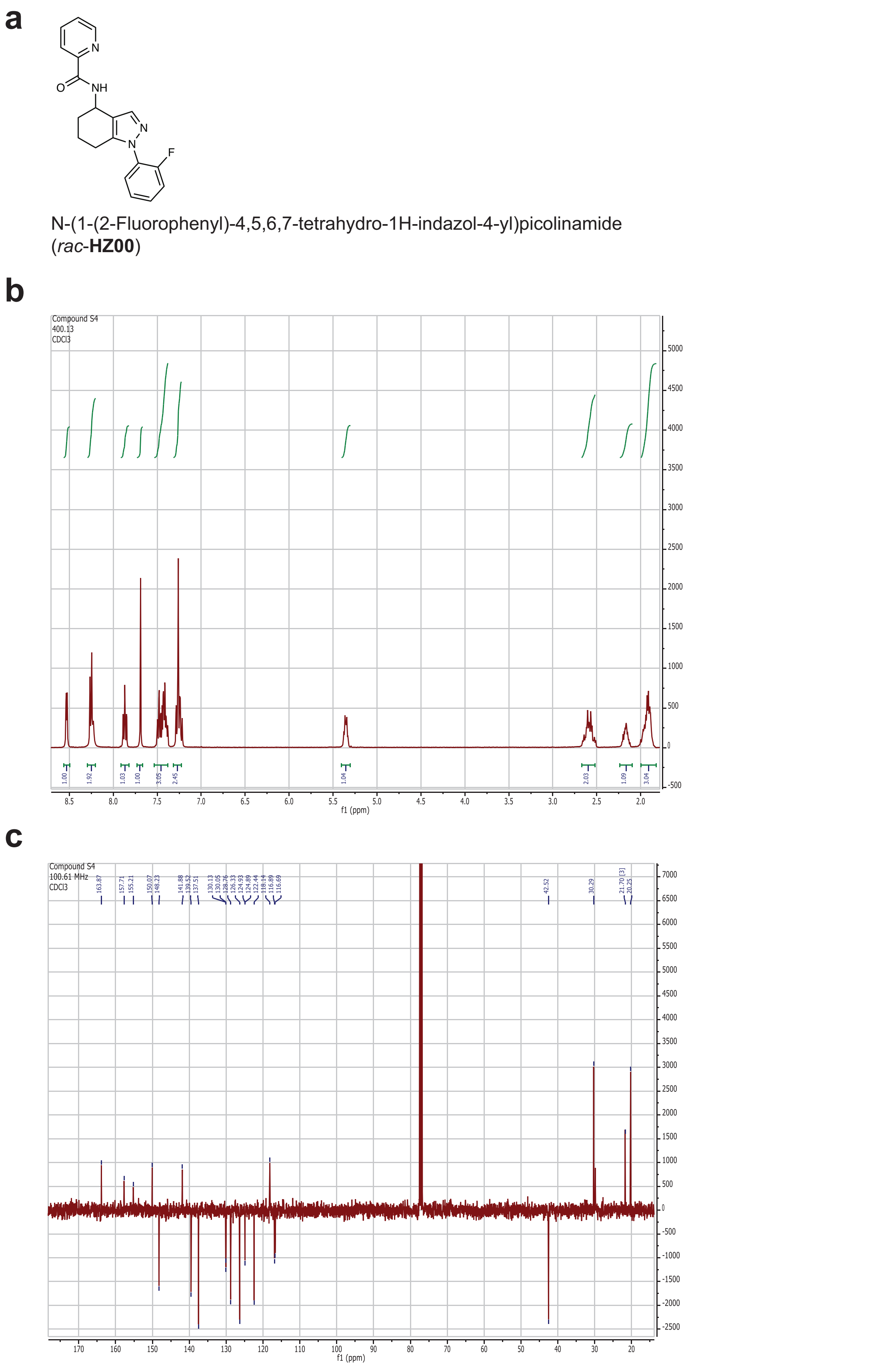

Supplementary Figure 15. Synthesis of *rac*-HZ00. a) Structure of *rac*-HZ00. b) ^{1}H-NMR trace of *rac*-HZ00. c) ^{13}C-NMR trace of *rac*-HZ00.

a

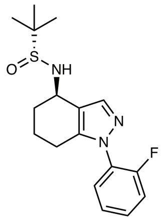

(S)-N-((R)-1-(2-Fluorophenyl)-4,5,6,7-tetrahydro-1H-indazol-4-yl)-2-methylpropane-2-sulfinamide
((*S*,*R*)-**2**)

b

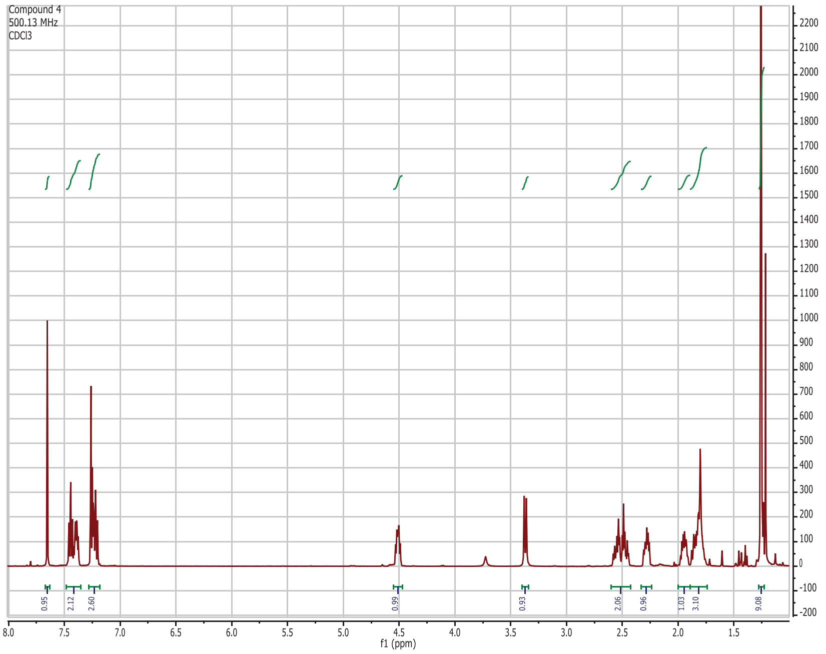

c

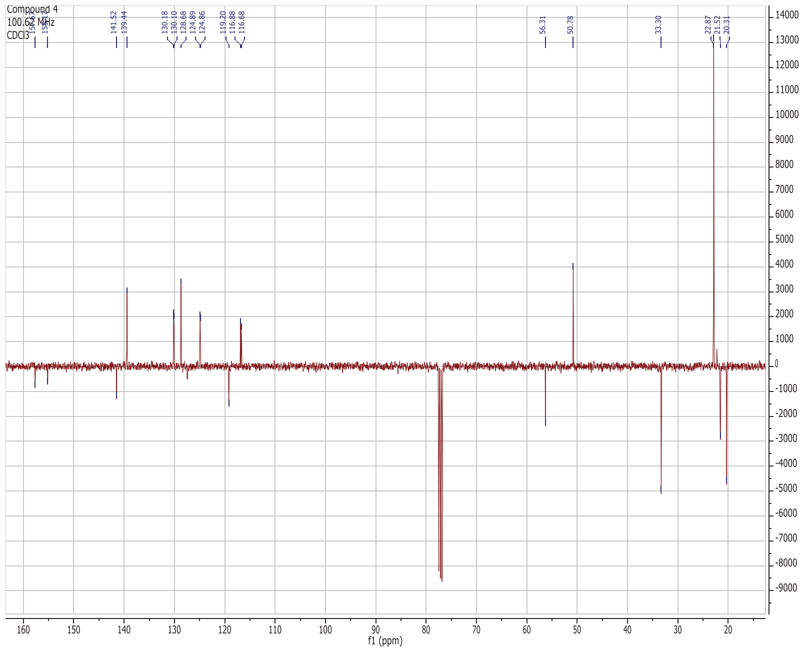

Supplementary Figure 16. Synthesis of (*S*,*R*)-2. a) Structure of (*S*,*R*)-2. b) ^{1}H-NMR trace of (*S*,*R*)-2. c) ^{13}C-NMR trace of (*S*,*R*)-2.

a

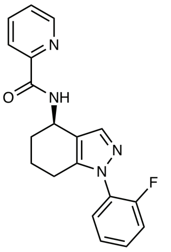

(R)-N-(1-(2-Fluorophenyl)-4,5,6,7-tetrahydro-1H-indazol-4-yl)picolinamide
((*R*)-HZ00)

b

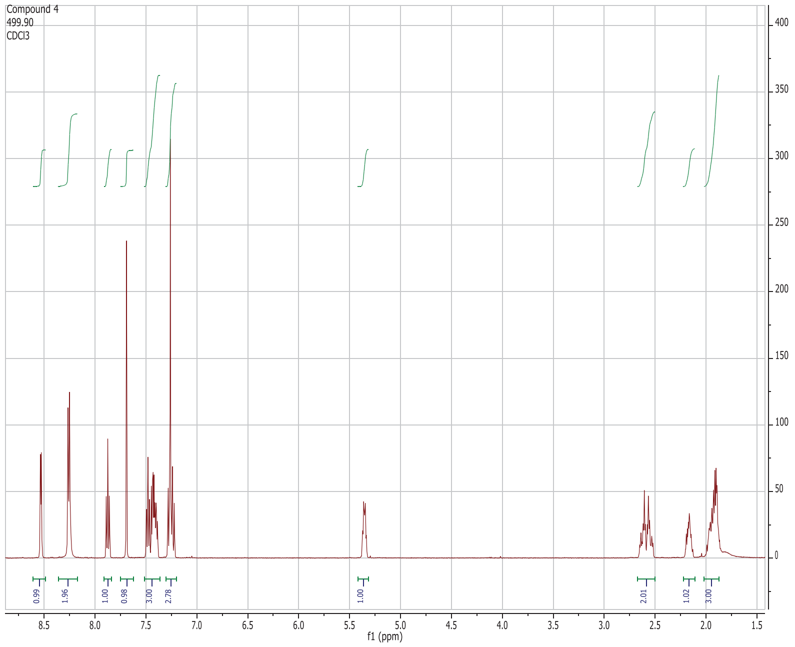

c

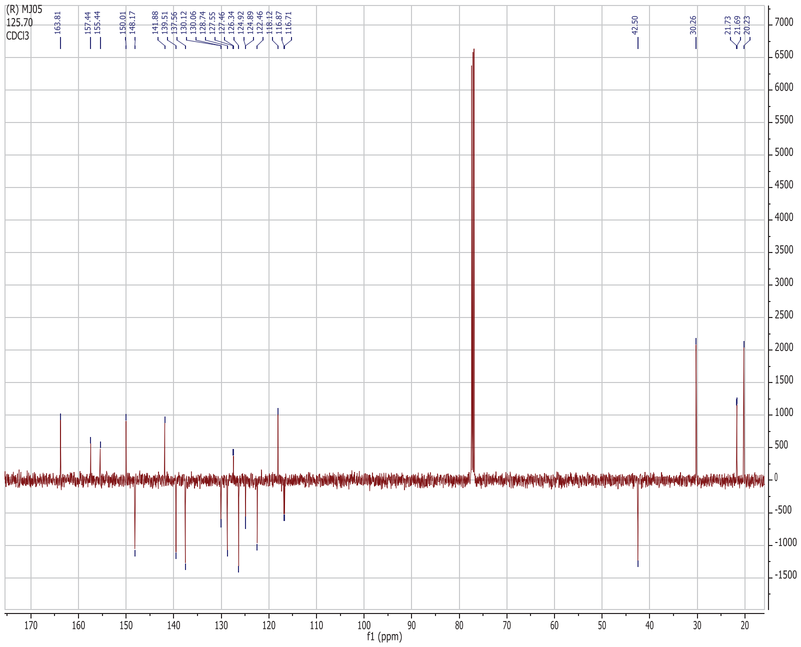

Supplementary Figure 17. Synthesis of (*R*)-HZ00. a) Structure of (*R*)-HZ00. b) ^{1}H-NMR trace of (*R*)-HZ00. c) ^{13}C-NMR trace of (*R*)-HZ00.

a

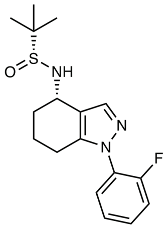

(S)-N-((S)-1-(2-Fluorophenyl)-4,5,6,7-tetrahydro-1H-indazol-4-yl)-2-methylpropane-2-sulfinamide ((*S,S*)-**2**)

b

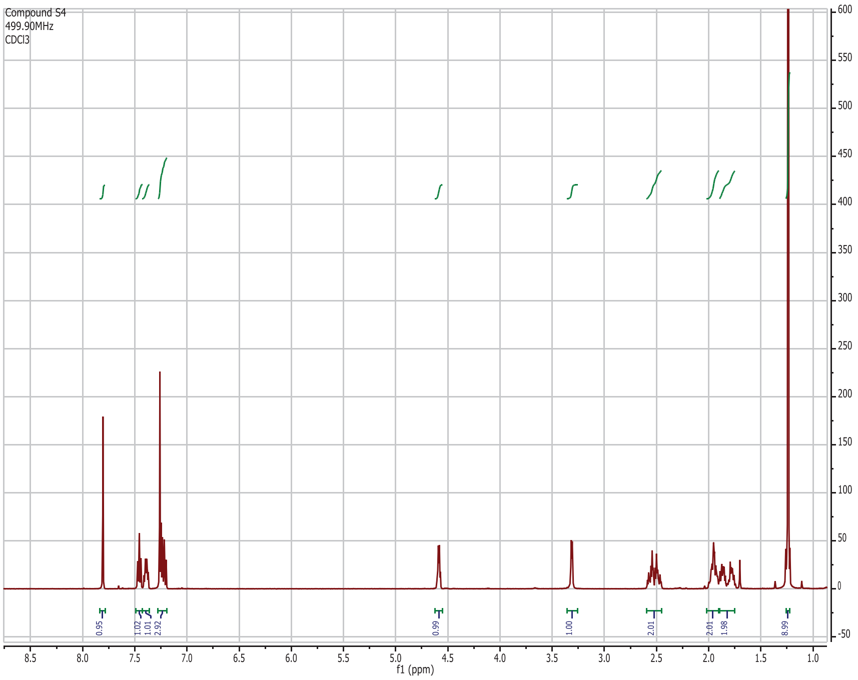

c

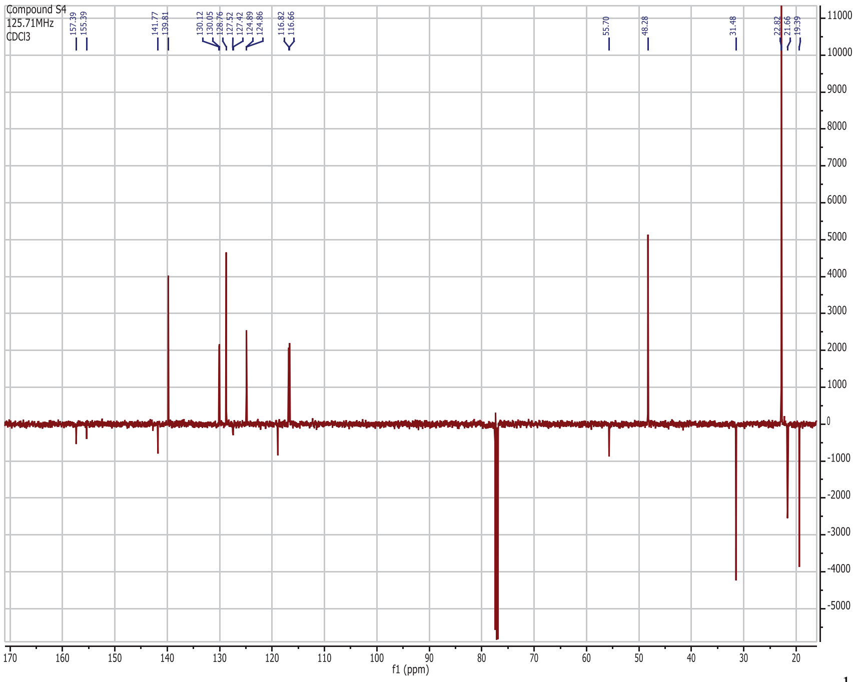

Supplementary Figure 18. Synthesis of (*S,S*)-2. a) Structure of (*S,S*)-2. b) ^{1}H-NMR trace of (*S,S*)-2. c) ^{13}C-NMR trace of (*S,S*)-2.

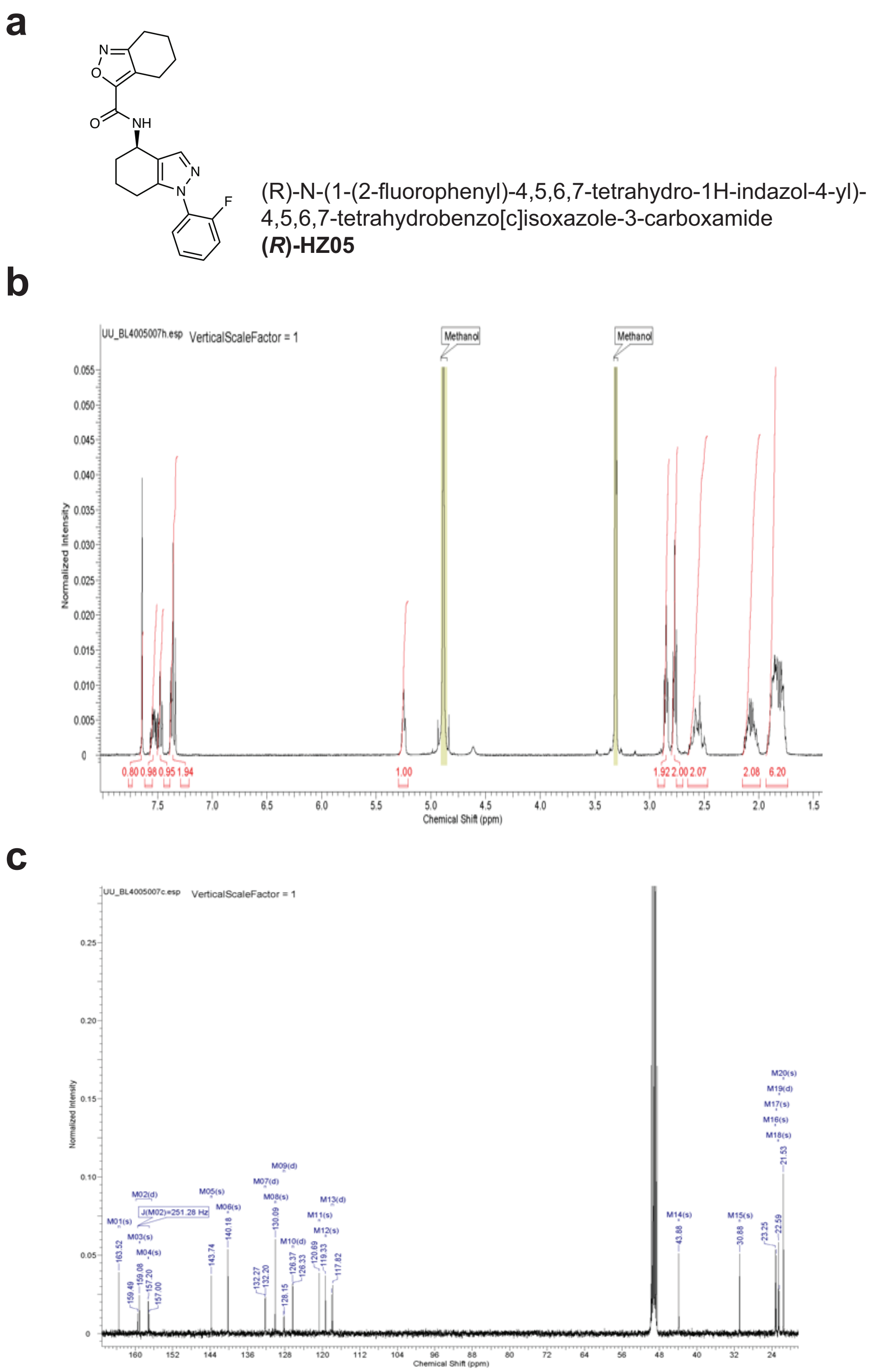

Supplementary Figure 19. Synthesis of (*R*)-HZ05. a) Structure of (*R*)-HZ05. b) ^{1}H-NMR trace of (*R*)-HZ05. c) ^{13}C-NMR trace of (*R*)-HZ05.

Supplementary Table 1. ***In vitro* inhibition (% remaining activity) of cyclin-dependent kinases (CDKs).**

HZ00 and (*R*)-HZ00 were screened against a panel of 16 cyclin-dependent kinases (CDKs) by ProQinase GmbH (Freiburg, Germany) using a radiometric protein kinase assay (33PanQinase® Activity Assay).

	**HZ00 10 µM**		**(*R*)-HZ00 10 µM**	
**Cyclin-Dependent Kinase**	**Average (% remaining activity)**	**St.Dev.**	**Average (% remaining activity)**	**St.Dev.**
CDK1/CycA2	89.1	8.4	90.9	2.5
CDK1/CycB1	103.6	1.7	100.1	4.1
CDK1/CycE1	101.3	7.3	103.0	3.1
CDK2/CycA2	96.0	7.2	100.7	3.6
CDK2/CycE1	101.7	6.0	96.8	2.4
CDK3/CycE1	96.6	7.2	90.8	3.1
CDK4/CycD1	103.9	5.6	104.0	1.8
CDK4/CycD3	89.2	3.1	98.3	2.1
CDK5/p25NCK	104.5	0.7	112.8	3.6
CDK5/p35NCK	105.2	0.3	101.1	8.4
CDK6/CycD1	102.7	12.0	97.1	7.5
CDK7/CycH/MAT1	96.2	2.0	105.4	3.5
CDK8/CycC	95.2	0.6	92.4	8.5
CDK9/CycK	100.3	19.2	95.9	8.9
CDK9/CycT1	100.2	3.1	106.6	3.1
PCTAIRE1/CycY	103.3	6.3	95.4	4.3

Supplementary Table 2. ***In vitro* inhibition (% remaining activity) of protein kinases.** HZ00 and (*R*)-HZ00 were screened against a panel of 140 kinases in cell-free assays. Screening was performed by the International Centre for Kinase Profiling (Dundee, UK) using radioactive (^{33}P-ATP) filter-binding assays.

	HZ00 10 µM		(*R*)-HZ00 10 µM	
protein kinase	Average	St.Dev.	Average	St.Dev.
ABL	100.0	6.1	97.0	1.4
AMPK	75.4	1.9	94.1	1.6
AMPK (hum)	113.2	4.0	100.3	0.8
ASK1	97.8	8.4	102.0	6.0
Aurora A	66.2	1.1	95.9	2.7
Aurora B	109.8	9.5	95.7	14.7
BRK	86.0	20.5	103.8	7.4
BRSK1	94.0	17.6	94.5	6.1
BRSK2	104.7	10.8	93.3	0.0
BTK	117.6	10.4	122.3	0.3
CAMK1	82.4	14.5	95.4	2.6
CAMKKb	75.9	2.7	95.6	4.3
CDK2-Cyclin A	95.8	18.3	101.1	4.1
CDK9-Cyclin T1	106.7	13.4	108.9	2.7
CHK1	102.4	8.9	102.5	4.7
CHK2	88.9	12.2	76.2	27.3
CK1γ2	117.5	6.7	97.6	14.6
CK1δ	95.3	0.1	91.1	7.8
CK2	111.4	0.2	110.6	9.1
CLK2	98.8	27.2	96.9	15.7
CSK	113.4	1.1	103.0	8.1
DAPK1	93.2	3.8	75.7	1.4
DDR2	87.6	0.9	100.4	3.0
DYRK1A	90.1	0.2	102.5	7.1
DYRK2	90.9	27.8	90.3	13.9
DYRK3	110.2	1.3	94.8	17.3
EF2K	94.1	1.0	100.1	6.1
EIF2AK3	99.0	5.0	103.3	2.3
EPH-A2	103.2	11.9	119.4	8.9
EPH-A4	85.6	9.4	106.4	12.1
EPH-B1	114.7	6.5	136.7	15.3
EPH-B2	83.2	6.9	124.9	31.3
EPH-B3	101.6	27.7	122.1	18.9
EPH-B4	107.1	6.6	125.7	13.8
ERK1	116.2	2.8	121.6	8.0
ERK2	116.8	3.8	109.0	1.5
ERK5	85.4	12.2	87.5	4.7
ERK8	106.9	11.9	98.9	0.1
FGF-R1	100.0	21.6	118.0	14.5
GCK	92.1	9.8	103.7	6.0
GSK3b	95.9	18.3	104.4	13.0
HER4	100.9	6.3	120.1	13.2
HIPK1	109.5	18.3	104.4	11.1
HIPK2	119.4	7.8	107.2	14.2
HIPK3	104.9	0.3	96.0	3.5
IGF-1R	149.9	9.8	134.6	13.9
IKKb	105.6	10.5	100.3	4.3

	HZ00 10 µM		(*R*)-HZ00 10 µM	
protein kinase	Average	St.Dev.	Average	St.Dev.
IKKe	75.4	0.3	110.2	20.8
IR	119.9	23.8	130.3	12.1
IRAK1	102.3	1.5	95.4	1.0
IRAK4	110.1	27.2	94.0	4.3
IRR	82.3	12.0	99.5	11.0
JAK2	101.7	16.5	103.4	10.6
JNK1	95.3	21.9	94.9	3.1
JNK2	90.4	9.0	105.9	8.8
JNK3	122.1	28.2	91.0	21.3
Lck	127.2	9.9	104.1	9.6
LKB1	126.8	19.7	103.1	8.0
MAP4K3	95.7	5.4	109.6	7.1
MAP4K5	109.5	2.9	119.6	21.0
MAPKAP-K2	95.4	4.1	92.2	6.9
MAPKAP-K3	88.2	7.8	97.8	2.5
MARK1	163.7	10.9	119.8	2.3
MARK2	87.3	18.0	90.2	1.8
MARK3	102.5	6.9	127.9	14.3
MARK4	79.0	10.0	95.2	16.8
MEKK1	97.6	3.3	110.5	1.2
MELK	100.7	5.8	90.4	10.4
MINK1	130.8	31.6	111.6	0.8
MKK1	78.3	10.6	102.9	8.6
MKK2	84.6	13.8	96.1	0.6
MKK6	83.8	9.3	83.9	0.1
MLK1	87.8	1.1	114.6	2.3
MLK3	120.1	6.6	100.4	2.8
MNK1	91.8	23.6	103.6	5.5
MNK2	97.9	1.4	91.5	8.5
MPSK1	69.2	8.3	96.5	24.6
MSK1	105.3	5.6	93.0	7.7
MST2	132.2	11.1	134.1	18.4
MST3	82.0	12.5	91.7	5.0
MST4	82.4	4.0	123.0	11.6
NEK2a	77.5	8.1	98.9	4.7
NEK6	78.8	4.8	101.8	0.0
NUAK1	83.5	0.7	102.1	7.3
OSR1	105.5	1.5	117.4	17.0
p38a MAPK	83.7	3.5	110.6	5.0
p38b MAPK	115.9	29.8	124.4	18.5
p38d MAPK	97.4	1.1	101.1	9.6
p38g MAPK	119.6	0.7	99.8	14.4
PAK2	110.5	7.8	100.5	5.3
PAK4	107.1	3.8	92.6	8.9
PAK5	111.7	2.4	87.9	1.5
PAK6	94.4	8.2	103.4	8.3
PDGFRA	98.1	12.6	105.8	5.3

	HZ00 10 µM		(*R*)-HZ00 10 µM	
protein kinase	Average	St.Dev.	Average	St.Dev.
PDK1	121.0	8.8	98.1	0.8
PHK	75.8	4.7	106.1	7.3
PIM1	75.4	8.4	100.6	23.7
PIM2	76.9	15.6	93.9	13.6
PIM3	91.5	0.3	108.4	10.5
PINK	109.8	1.1	87.9	10.5
PKA	77.7	3.6	89.0	7.1
PKBa	106.9	1.1	111.5	5.1
PKBb	101.3	3.4	118.4	11.0
PKCa	80.1	1.8	98.9	7.6
PKCz	79.9	7.4	113.5	3.3
PKCγ	85.4	16.2	102.9	5.7
PKD1	114.8	34.8	128.1	20.8
PLK1	118.8	4.5	108.7	1.4
PRAK	95.7	4.7	78.4	0.5
PRK2	106.1	12.2	109.7	1.5
RIPK2	98.3	22.1	99.1	0.1
ROCK 2	127.2	3.5	89.1	8.0
RSK1	111.0	0.3	99.1	0.0
RSK2	92.5	7.0	83.3	11.1
S6K1	103.8	4.3	94.4	6.0
SGK1	114.4	19.3	98.9	11.4
SIK2	95.9	11.6	96.7	18.3
SIK3	89.6	9.2	99.8	1.0
SmMLCK	91.7	8.6	95.9	1.4
Src	110.0	2.5	97.4	1.5
SRPK1	76.0	16.0	107.8	28.3
STK33	93.1	9.0	107.2	7.5
SYK	115.6	21.2	101.8	8.1
TAK1	87.5	2.6	115.6	19.4
TAO1	85.4	36.4	101.1	1.1
TBK1	101.0	10.5	102.1	21.6
TESK1	106.7	16.1	113.1	2.2
TGFBR1	113.0	7.7	98.8	5.8
TIE2	89.2	0.1	98.5	1.7
TLK1	99.2	2.5	98.6	8.4
TrkA	108.7	3.3	102.0	2.8
TSSK1	81.7	2.3	96.1	0.0
TTBK1	79.2	6.3	100.3	26.5
TTBK2	97.0	9.2	100.1	7.5
TTK	113.9	3.7	105.2	3.9
ULK1	86.4	9.8	105.0	11.4
ULK2	84.9	0.3	112.0	16.3
VEG-FR	116.5	3.8	98.9	13.3
WNK1	96.9	30.2	96.2	16.3
YES1	97.4	1.0	111.4	9.5
ZAP70	108.3	25.8	105.9	16.8

Supplementary Table 3. *In vitro* inhibition (% remaining activity) of lipid kinases. HZ00 and (*R*)-HZ00 were screened *in vitro* against a panel of 16 lipid kinases using ADP-Glo™ kinase assays (Promega) according to manufacturer's instructions (International Centre for Kinase Profiling, Dundee, UK).

	**HZ00 10 µM**		**(*R*)-HZ00 10 µM**	
**Lipid Kinase**	**Average (% remaining activity)**	**St.Dev.**	**Average (% remaining activity)**	**St.Dev.**
Choline Kinase Alpha	104.4	8.86	101.7	11.47
Choline Kinase beta	99.3	13	93.9	16.58
DGK beta	105.1	27.1	114.9	21.36
DGK gamma	94.3	16.07	109	3.15
DGK zeta	94.1	26.16	95.7	36.67
PI3 kinase alpha	96	15.32	91.8	24.46
PI3 Kinase alpha E542K	94.3	10.24	94.5	24.81
PI3 Kinase alpha E545K	102.9	4.81	105.7	28.15
PI3 kinase beta	94.4	2.4	94.3	21.73
PI3 kinase delta	103.5	11.34	94.5	14.89
PI3 kinase gamma	97.7	11.68	99.7	6.38
PI4K2A	114.7	3.3	106.2	10.62
PIP5K2A	95.1	8.81	98.3	10
Sphingosine kinase 1	85.8	8.99	83.3	13.8
Sphingosine kinase 2	91.3	6.01	79	10.16

Supplementary Table 4. *In vitro* preclinical properties of HZ00. Plasma protein binding and stability was determined in pooled human plasma and fraction unbound and percentage stability calculated following LC-MS/MS analysis. The metabolic stability of HZ00 in human liver microsomes was determined and results used to calculate *in vitro* half-life ($t_{1/2}$) and *in vitro* clearance (C_{lint}). Caco-2 permeability studies were used to investigate membrane permeability and efflux ratio calculated.

Chemical Stability	% after 5 h at pH 7.4	100
Plasma Protein Binding and Stability	Fraction unbound (%)	9.3 ± 0.9
	Stability (%)	103 ± 3
Metabolic Stability	In vitro $t_{1/2}$ (min)	67
	Clint (μL/min x mg)	21
Caco-2 Permeability	Papp a-b (x10^6 cm/s)	109 ± 15
	Papp b-a (x10^6 cm/s)	130 ± 9
	Efflux Ratio (ba/ab)	1.2

Supplementary Table 5. DHODH inhibition by compounds (at 10 μM) from the 20,000 compound library (ChemBridge) that activate p53 at 10 μM in ARN8 melanoma cells in the primary screen (HZ00 is not included). These compounds were then tested for p53 activation at different concentrations in ARN8 and in T22 cells. Compounds that activated p53-dependent transcription more than 1.5 fold in ARN8 cells at any of the concentrations tested are shaded in yellow, compounds that activated p53-dependent transcription more than 1.5 fold in in T22 cells are shaded in blue, compounds that activated p53-dependent transcription more than 1.5 fold in both cell lines are shaded in green and compounds that activated p53 less than 1.5 fold in both cell lines are not shaded. * Compounds that include substructure elements reported in PAIN compounds.

ChemBridge ID	Compound name	remaining DHODH activity (%)		Chemotype family
		Average	StDev	
7723007	2-({[(4-methylbenzyl)thio]acetyl}amino)benzoic acid	-8.22	3.62	Bisarylamides
51157786	2-[(2-isopropyl-1,3-thiazol-4-yl)carbonyl]-7-(2-phenylethyl)-2,7-diazaspiro[4.5]decane	-5.48	1.37	Singleton
55410102	N-(sec-butyl)-5-[3-(2,3-dimethoxyphenyl)-1,2,4-oxadiazol-5-yl]-2-pyridinamine	2.28	2.09	Aryloxadiazoles
7841649	2-methyl-N-(4-methyl-2-oxo-2H-chromen-7-yl)-1-indolinecarbothioamide	3.65	2.09	Chromen-2-ones
75901793	(1S*,4S*)-2-{5-[3-(2,3-dimethoxyphenyl)-1,2,4-oxadiazol-5-yl]-2-pyridinyl}-2-azabicyclo[2.2.1]heptane	5.94	0.79	Aryloxadiazoles
54631106	((2S)-1-{5-[3-(2-methylphenyl)-1,2,4-oxadiazol-5-yl]-2-pyridinyl}-2-pyrrolidinyl)methanol	5.94	0.79	Aryloxadiazoles
57235883	5-[3-(2,3-dimethoxyphenyl)-1,2,4-oxadiazol-5-yl]-N,N-diethyl-2-pyridinamine	9.40	1.96	Aryloxadiazoles
47870602	2-(4-methoxy-3-methylphenyl)-4-{[2-(2-methoxyphenyl)-1-pyrrolidinyl]methyl}-5-methyl-1,3-oxazole	10.68	1.96	Aryloxazoles
7690424	2-(1H-indol-3-yl)-2-oxo-N-(4,5,6,7-tetrahydro-1,3-benzothiazol-2-yl)acetamide	10.96	1.37	Bisarylamides
57999135	5-[3-(2,3-dimethoxyphenyl)-1,2,4-oxadiazol-5-yl]-2-(1-piperidinyl)pyridine	16.67	1.96	Aryloxadiazoles
5135537	N-2-naphthyl-1-adamantanecarboxamide	31.96	2.09	Singleton
78928129	1-{5-[3-(2,3-dimethoxyphenyl)-1,2,4-oxadiazol-5-yl]-2-pyridinyl}-3-piperidinol	42.31	1.48	Aryloxadiazoles
6592864	5-bromo-N-{[(2-methylphenyl)amino]carbonothioyl}-1-naphthamide	72.15	2.85	Phenylcarbamothioylbenzamides
7638934	N-(2-ethyl-6-methylphenyl)-2-{[3-(1-naphthyl)-4-oxo-3,4-dihydro-2-quinazolinyl]thio}acetamide	72.65	3.39	Quinazolinones
7782544	N-(4-hydroxy-2-methylphenyl)-4-biphenylcarboxamide	73.50	4.50	Bisarylamides
5351720	N-(4-ethylphenyl)-5-nitro-2-furamide	73.52	2.09	Bisarylamides
7776857	2-{3-[2-(2-thienyl)vinyl]-1H-1,2,4-triazol-5-yl}phenol	77.35	5.34	Aryloxadiazoles
7578012*	N-({[4-(diethylamino)phenyl]amino}carbonothioyl)-2-methoxy-3-methylbenzamide	79.00	0.79	Phenylcarbamothioylbenzamides
7574753	(3-chloro-4,5-dimethoxybenzyl)(3-chloro-4-methylphenyl)amine	79.91	0.79	Bisarylamines
5264537*	ethyl 4-phenyl-2-[(3,4,5-trimethoxybenzoyl)amino]-3-thiophenecarboxylate	82.91	3.39	Bisarylamides
5356839	N-(5-chloro-2-methoxyphenyl)-5-nitro-2-furamide	83.76	0.74	Bisarylamides
47561416	1-{[5-methyl-2-(5-methyl-2-furyl)-1,3-oxazol-4-yl]methyl}-2-(1,3-thiazol-2-yl)piperidine	86.30	0.00	Aryloxadiazoles
7800701	9-(2-isopropoxyphenyl)-2,3,8,9-tetrahydro[1,4]dioxino[2,3-g]quinolin-7(6H)-one	88.13	5.19	Phenyl-dihydroquinolinones
76026685	1-(2-fluoro-4-methoxyphenyl)-2-(3-furylmethyl)-2,3,4,9-tetrahydro-1H-beta-carboline	88.46	4.85	Tetrahydrocarbazoles
5351342	N-(2,5-dichlorophenyl)-5-nitro-2-furamide	90.41	6.28	Bisarylamides
90739158	1-(4-methoxy-3-methylphenyl)-2-(1,3-thiazol-2-ylmethyl)-2,3,4,9-tetrahydro-1H-beta-carboline	91.88	2.56	Tetrahydrocarbazoles
7794325	2-[({[2-(trifluoromethyl)phenyl]amino}carbonyl)amino]benzamide	92.24	2.09	Singleton
7587351	2-[(8-ethoxy-4-methyl-2-quinazolinyl)amino]-5,6,7,8-tetrahydro-4(1H)-quinazolinone	92.69	2.85	Quinazolinones
6912406	N-[1-(2,5-dimethoxyphenyl)ethyl]-2-furamide	93.15	2.37	Bisarylamides
7706732	ethyl 7-(2-furyl)-2-methyl-5-oxo-4-(2-thienyl)-1,4,5,6,7,8-hexahydro-3-quinolinecarboxylate	95.89	0.00	Tetrahydroquinolinones
6623100	3-(6-chloroimidazo[1,2-a]pyridin-2-yl)-2H-chromen-2-one	96.35	0.79	Chromen-2-ones
7706999	2-{2-[3-ethoxy-4-(propionyloxy)phenyl]vinyl}-8-quinolinyl propionate	98.17	1.58	Singleton
7739721	N-[3,5-bis(trifluoromethyl)phenyl]-2,3,4,5,6-pentafluorobenzamide	98.17	2.09	Bisarylamides
7801595	methyl 3-({[(5-chloro-2-methoxyphenyl)amino]carbonothioyl}amino)benzoate	100.46	3.45	Singleton
7834586	9-[2-(benzyloxy)phenyl]-2,3,8,9-tetrahydro[1,4]dioxino[2,3-g]quinolin-7(6H)-one	102.56	1.48	Phenyl-dihydroquinolinones
7617239	2-{[2-(1,3-benzothiazol-2-ylsulfonyl)ethyl]thio}-1,3-benzoxazole	103.20	8.91	Bisarylsulphonamides
7705624	4-[2-(benzyloxy)phenyl]-N-(4-fluorophenyl)-2-methyl-5-oxo-1,4,5,6,7,8-hexahydro-3-quinolinecarboxamide	104.27	0.74	Tetrahydroquinolinones

Supplementary Table 6. DHODH inhibition by compounds (at 10 μM) from the 30,000 compound library (ChemBridge) that activate p53 at 5 μM in ARN8 melanoma cells in the primary screen. These compounds were then tested for p53 activation at different concentrations in ARN8 and searched for amongst compounds that were active in T22 cells in a previous screen of the same library[13]. Color code is as in supplementary table 5. * Compounds that include substructure elements reported in PAIN compounds.

ChemBridge ID	Compound name	remaining DHODH activity (%)		Chemotype family
		Average	StDev	
5248918*	2-(4-biphenylyl)-3-hydroxynaphthoquinone	-1.54	12.74	Quinone
5347717	N'-(4-hydroxybenzylidene)-2-phenyl-4-quinolinecarbohydrazide	8.96	11.24	Hydrazones
5303009	N-cyclohexyl-6-methyl-2,3,4,9-tetrahydro-1H-carbazol-1-amine	12.59	6.67	Tetrahydrocarbazoles
5120580	1-(4-biphenylyl)-2-(2-imino-3-methyl-1(2H)-pyridinyl)ethanone hydrobromide	19.22	5.93	Singleton
5869604	N-benzyl-6-methyl-2,3,4,9-tetrahydro-1H-carbazol-1-amine hydrochloride	27.30	0.84	Tetrahydrocarbazoles
5561538	(3-bromo-4-methoxybenzyl)(4-bromo-3-methylphenyl)amine	42.50	7.53	Bisarylamines
5232020	2-(2-naphthyl)-2,3,5,6,7,8-hexahydro[1]benzothieno[2,3-d]pyrimidin-4(1H)-one	43.45	7.54	Thienopyrimidinones
5666718	3-[(2,3,4,9-tetrahydro-1H-carbazol-1-ylamino)methylene]-1,3-dihydro-2H-indol-2-one	48.47	1.74	Tetrahydrocarbazoles
6611243	4-tert-butyl-N-{[(2-hydroxy-4,5-dimethylphenyl)amino]carbonothioyl}benzamide	50.97	2.69	Phenylcarbamothioylbenzamides
5781453	N-[2-(2-methoxyphenyl)-1-methylethyl]-6-nitro-2,3,4,9-tetrahydro-1H-carbazol-1-amine	52.37	0.84	Tetrahydrocarbazoles
5781454	N-[2-(2-fluorophenyl)-1-methylethyl]-6-nitro-2,3,4,9-tetrahydro-1H-carbazol-1-amine	55.43	6.05	Tetrahydrocarbazoles
5265927	6-bromo-N-(2-phenylethyl)-2,3,4,9-tetrahydro-1H-carbazol-1-amine hydrochloride	55.71	3.64	Tetrahydrocarbazoles
5781447	N-(4-methylcyclohexyl)-6-nitro-2,3,4,9-tetrahydro-1H-carbazol-1-amine	56.82	1.93	Tetrahydrocarbazoles
5781444	N-(2,3-dihydro-1H-inden-2-yl)-6-nitro-2,3,4,9-tetrahydro-1H-carbazol-1-amine	57.94	3.38	Tetrahydrocarbazoles
5373779	6-bromo-2,3,4,9-tetrahydro-1H-carbazol-1-one hydrazone	58.19	10.15	Tetrahydrocarbazoles/Hydrazones
5781441	6-nitro-N-[1-(2-phenylethyl)-4-piperidinyl]-2,3,4,9-tetrahydro-1H-carbazol-1-amine	59.61	0.96	Tetrahydrocarbazoles
5781446	N-(3-methylcyclopentyl)-6-nitro-2,3,4,9-tetrahydro-1H-carbazol-1-amine	60.45	1.74	Tetrahydrocarbazoles
6519439	N-({[3-(benzoylamino)phenyl]amino}carbonothioyl)-4-tert-butylbenzamide	63.23	1.67	Phenylcarbamothioylbenzamides
6641022	2-naphthyl(2-phenoxyethyl)amine	66.02	2.69	Bisarylamines
5650027	2,2,2-trichloro-1-(9-methyl-2-phenyl-9H-imidazo[1,2-a]benzimidazol-3-yl)ethanol	68.38	12.02	Benzimidazol
5705453	2-[2-(5-methoxy-1,2-dimethyl-1H-indol-3-yl)vinyl]-4(3H)-quinazolinone	66.14	1.20	Quinazolinones
5704541	N-1,3-benzothiazol-2-yl-9H-xanthene-9-carboxamide	71.03	2.10	Bisarylamides
6638197	4-[4-(benzyloxy)benzylidene]-2-(methylthio)-1,3-thiazol-5(4H)-one	74.93	1.45	Thiazol-like
5554099	2-(2-bromophenoxy)-N'-(2,4-dimethylbenzylidene)acetohydrazide	78.09	1.20	Hydrazones
5781442	N-[2-(3,4-dimethoxyphenyl)-1-methylethyl]-6-nitro-2,3,4,9-tetrahydro-1H-carbazol-1-amine	78.27	1.67	Tetrahydrocarbazoles
6638116	4-[4-(benzyloxy)-3-bromo-5-methoxybenzylidene]-2-(methylthio)-1,3-thiazol-5(4H)-one	78.83	4.90	Thiazol-like
5705506*	2-{2-[4-(dimethylamino)phenyl]vinyl}-4(3H)-quinazolinone	82.47	3.84	Quinazolinones
6501326	4-tert-butyl-N-{[(4-ethoxyphenyl)amino]carbonothioyl}benzamide	84.12	3.64	Phenylcarbamothioylbenzamides
6515740	4-tert-butyl-N-{[(3-nitrophenyl)amino]carbonothioyl}benzamide	88.86	1.28	Phenylcarbamothioylbenzamides
5781470	N-isopropyl-6-nitro-2,3,4,9-tetrahydro-1H-carbazol-1-amine	90.25	0.96	Tetrahydrocarbazoles
5213777	3-(1,3-benzoxazol-2-yl)-7-(diethylamino)-2H-chromen-2-one	92.14	1.64	Chromen-2-ones
5666613	5,5-dimethyl-2-{[(6-methyl-2,3,4,9-tetrahydro-1H-carbazol-1-yl)amino]methylene}-1,3-cyclohexanedione	92.20	4.75	Tetrahydrocarbazoles
5313702	N'-(2-bromo-3-phenyl-2-propen-1-ylidene)-1,4,5,6-tetrahydrocyclopenta[c]pyrazole-3-carbohydrazide	92.49	0.68	Hydrazones
5213776	7-(diethylamino)-3-(1-methyl-1H-benzimidazol-2-yl)-2H-chromen-2-one	96.79	1.24	Chromen-2-ones
5754791	N-(3-acetyl-7-bromo-2-methyl-1-benzofuran-5-yl)-4-ethoxybenzenesulfonamide	96.81	0.69	Bisarylsulphonamides
5276937	1-(4-chloro-3-nitrobenzoyl)-4-(2-methoxyphenyl)piperazine	97.50	1.64	Bisarylamides
5852371	4-bromo-N-[5-(3-methylbenzyl)-1,3-thiazol-2-yl]benzamide	97.61	1.38	Bisarylamides
5321611	2-[(3-chlorobenzylidene)amino]benzamide	97.63	2.05	Benzylideneaminobenzamides
6513572	4-tert-butyl-N-{[(2,4-dimethoxyphenyl)amino]carbonothioyl}benzamide	97.77	1.74	Phenylcarbamothioylbenzamides
5545710	3-nitro-N'-(3-phenoxybenzylidene)benzohydrazide	98.02	4.16	Hydrazones
5625039	2-(2-phenylvinyl)-5,6,7,8-tetrahydro[1]benzothieno[2,3-d]pyrimidin-4(3H)-one	98.41	3.59	Thienopyrimidinones
5472837	N'-{[5-(4-bromophenyl)-2-furyl]methylene}-2-(4-methylphenoxy)acetohydrazide	98.42	2.47	Hydrazones
5267938	2,2'-[3,6-acridinediylbis(nitrilomethylylidene)]diphenol	98.57	0.62	Singleton
5344759	4,5-dichloro-2-[4-chloro-3-(trifluoromethyl)phenyl]-3(2H)-pyridazinone	98.81	0.00	Singleton
5284467*	5-bromo-3-[3-(2-methoxyphenyl)-4-oxo-2-thioxo-1,3-thiazolidin-5-ylidene]-1,3-dihydro-2H-indol-2-one	98.93	2.47	Thiazol-like
5867214	N-(2-methoxyphenyl)-9H-xanthene-9-carboxamide	99.16	1.45	Bisarylamides
5351654	N-(4-iodo-2-methylphenyl)-4-methoxybenzenesulfonamide	99.21	2.47	Bisarylsulphonamides
6149306	3-chloro-4-[(5-chloro-2-methoxyphenyl)amino]-1-(2,3-dimethylphenyl)-1H-pyrrole-2,5-dione	99.60	2.39	Singleton
5301107*	N-(5-amino-9,10-dioxo-9,10-dihydro-1-anthracenyl)benzamide	100.00	3.27	Bisarylamides
5378429	6-chloro-2-methyl-N'-(4-methylbenzylidene)imidazo[1,2-a]pyridine-3-carbohydrazide	100.00	1.19	Hydrazones
5220809	3-[(2-methoxyphenyl)amino]-5-phenyl-2-cyclohexen-1-one	100.00	0.62	Bisarylamines
5249701	N'-(4-bromobenzylidene)-2,3-dihydro-1,4-benzodioxine-2-carbohydrazide	100.00	0.62	Hydrazones
6497060*	4-isopropoxy-N-({[4-(1-piperidinyl)phenyl]amino}carbonothioyl)benzamide	100.84	0.84	Phenylcarbamothioylbenzamides
5175570	2-methoxyethyl 5,6,7,8-tetrahydro[1]benzothieno[2,3-d][1,3]thiazol-2-ylcarbamate	101.07	2.23	Singleton
5783994	4-benzyl-N-(2,4-dimethoxybenzylidene)-1-piperazinamine	101.11	1.28	Hydrazones
5373649	3,4-dihydro-2(1H)-quinolinone phenylhydrazone hydrochloride	101.19	1.19	Hydrazones
5320101	2-[(2-methylbenzylidene)amino]benzamide	101.19	0.00	Benzylideneaminobenzamides

Table 6 continued

ChemBridge ID	Compound name	remaining DHODH activity (%)		Chemotype family
		Average	StDev	
5248918*	2-(4-biphenylyl)-3-hydroxynaphthoquinone	-1.54	12.74	Quinone
5347717	N'-(4-hydroxybenzylidene)-2-phenyl-4-quinolinecarbohydrazide	8.96	11.24	Hydrazones
5303009	N-cyclohexyl-6-methyl-2,3,4,9-tetrahydro-1H-carbazol-1-amine	12.59	6.67	Tetrahydrocarbazoles
5120580	1-(4-biphenylyl)-2-(2-imino-3-methyl-1(2H)-pyridinyl)ethanone hydrobromide	19.22	5.93	Singleton
5869604	N-benzyl-6-methyl-2,3,4,9-tetrahydro-1H-carbazol-1-amine hydrochloride	27.30	0.84	Tetrahydrocarbazoles
5561538	(3-bromo-4-methoxybenzyl)(4-bromo-3-methylphenyl)amine	42.50	7.53	Bisarylamines
5232020	2-(2-naphthyl)-2,3,5,6,7,8-hexahydro[1]benzothieno[2,3-d]pyrimidin-4(1H)-one	43.45	7.54	Thienopyrimidinones
5666718	3-[(2,3,4,9-tetrahydro-1H-carbazol-1-ylamino)methylene]-1,3-dihydro-2H-indol-2-one	48.47	1.74	Tetrahydrocarbazoles
6611243	4-tert-butyl-N-{[(2-hydroxy-4,5-dimethylphenyl)amino]carbonothioyl}benzamide	50.97	2.69	Phenylcarbamothioylbenzamides
5781453	N-[2-(2-methoxyphenyl)-1-methylethyl]-6-nitro-2,3,4,9-tetrahydro-1H-carbazol-1-amine	52.37	0.84	Tetrahydrocarbazoles
5781454	N-[2-(2-fluorophenyl)-1-methylethyl]-6-nitro-2,3,4,9-tetrahydro-1H-carbazol-1-amine	55.43	6.05	Tetrahydrocarbazoles
5265927	6-bromo-N-(2-phenylethyl)-2,3,4,9-tetrahydro-1H-carbazol-1-amine hydrochloride	55.71	3.64	Tetrahydrocarbazoles
5781447	N-(4-methylcyclohexyl)-6-nitro-2,3,4,9-tetrahydro-1H-carbazol-1-amine	56.82	1.93	Tetrahydrocarbazoles
5781444	N-(2,3-dihydro-1H-inden-2-yl)-6-nitro-2,3,4,9-tetrahydro-1H-carbazol-1-amine	57.94	3.38	Tetrahydrocarbazoles
5373779	6-bromo-2,3,4,9-tetrahydro-1H-carbazol-1-one hydrazone	58.19	10.15	Tetrahydrocarbazoles/Hydrazones
5781441	6-nitro-N-[1-(2-phenylethyl)-4-piperidinyl]-2,3,4,9-tetrahydro-1H-carbazol-1-amine	59.61	0.96	Tetrahydrocarbazoles
5781446	N-(3-methylcyclopentyl)-6-nitro-2,3,4,9-tetrahydro-1H-carbazol-1-amine	60.45	1.74	Tetrahydrocarbazoles
6519439	N-({[3-(benzoylamino)phenyl]amino}carbonothioyl)-4-tert-butylbenzamide	63.23	1.67	Phenylcarbamothioylbenzamides
6641022	2-naphthyl(2-phenoxyethyl)amine	66.02	2.69	Bisarylamines
5650027	2,2,2-trichloro-1-(9-methyl-2-phenyl-9H-imidazo[1,2-a]benzimidazol-3-yl)ethanol	68.38	12.02	Benzimidazol
5705453	2-[2-(5-methoxy-1,2-dimethyl-1H-indol-3-yl)vinyl]-4(3H)-quinazolinone	66.14	1.20	Quinazolinones
5704541	N-1,3-benzothiazol-2-yl-9H-xanthene-9-carboxamide	71.03	2.10	Bisarylamides
6638197	4-[4-(benzyloxy)benzylidene]-2-(methylthio)-1,3-thiazol-5(4H)-one	74.93	1.45	Thiazol-like
5554099	2-(2-bromophenoxy)-N'-(2,4-dimethylbenzylidene)acetohydrazide	78.09	1.20	Hydrazones
5781442	N-[2-(3,4-dimethoxyphenyl)-1-methylethyl]-6-nitro-2,3,4,9-tetrahydro-1H-carbazol-1-amine	78.27	1.67	Tetrahydrocarbazoles
6638116	4-[4-(benzyloxy)-3-bromo-5-methoxybenzylidene]-2-(methylthio)-1,3-thiazol-5(4H)-one	78.83	4.90	Thiazol-like
5705506*	2-{2-[4-(dimethylamino)phenyl]vinyl}-4(3H)-quinazolinone	82.47	3.84	Quinazolinones
6501326	4-tert-butyl-N-{[(4-ethoxyphenyl)amino]carbonothioyl}benzamide	84.12	3.64	Phenylcarbamothioylbenzamides
6515740	4-tert-butyl-N-{[(3-nitrophenyl)amino]carbonothioyl}benzamide	88.86	1.28	Phenylcarbamothioylbenzamides
5781470	N-isopropyl-6-nitro-2,3,4,9-tetrahydro-1H-carbazol-1-amine	90.25	0.96	Tetrahydrocarbazoles
5213777	3-(1,3-benzoxazol-2-yl)-7-(diethylamino)-2H-chromen-2-one	92.14	1.64	Chromen-2-ones
5666613	5,5-dimethyl-2-{[(6-methyl-2,3,4,9-tetrahydro-1H-carbazol-1-yl)amino]methylene}-1,3-cyclohexanedione	92.20	4.75	Tetrahydrocarbazoles
5313702	N'-(2-bromo-3-phenyl-2-propen-1-ylidene)-1,4,5,6-tetrahydrocyclopenta[c]pyrazole-3-carbohydrazide	92.49	0.68	Hydrazones
5213776	7-(diethylamino)-3-(1-methyl-1H-benzimidazol-2-yl)-2H-chromen-2-one	96.79	1.24	Chromen-2-ones
5754791	N-(3-acetyl-7-bromo-2-methyl-1-benzofuran-5-yl)-4-ethoxybenzenesulfonamide	96.81	0.69	Bisarylsulphonamides
5276937	1-(4-chloro-3-nitrobenzoyl)-4-(2-methoxyphenyl)piperazine	97.50	1.64	Bisarylamides
5852371	4-bromo-N-[5-(3-methylbenzyl)-1,3-thiazol-2-yl]benzamide	97.61	1.38	Bisarylamides
5321611	2-[(3-chlorobenzylidene)amino]benzamide	97.63	2.05	Benzylideneaminobenzamides
6513572	4-tert-butyl-N-{[(2,4-dimethoxyphenyl)amino]carbonothioyl}benzamide	97.77	1.74	Phenylcarbamothioylbenzamides
5545710	3-nitro-N'-(3-phenoxybenzylidene)benzohydrazide	98.02	4.16	Hydrazones
5625039	2-(2-phenylvinyl)-5,6,7,8-tetrahydro[1]benzothieno[2,3-d]pyrimidin-4(3H)-one	98.41	3.59	Thienopyrimidinones
5472837	N'-{[5-(4-bromophenyl)-2-furyl]methylene}-2-(4-methylphenoxy)acetohydrazide	98.42	2.47	Hydrazones
5267938	2,2'-[3,6-acridinediylbis(nitrilomethylylidene)]diphenol	98.57	0.62	Singleton
5344759	4,5-dichloro-2-[4-chloro-3-(trifluoromethyl)phenyl]-3(2H)-pyridazinone	98.81	0.00	Singleton
5284467*	5-bromo-3-[3-(2-methoxyphenyl)-4-oxo-2-thioxo-1,3-thiazolidin-5-ylidene]-1,3-dihydro-2H-indol-2-one	98.93	2.47	Thiazol-like
5867214	N-(2-methoxyphenyl)-9H-xanthene-9-carboxamide	99.16	1.45	Bisarylamides
5351654	N-(4-iodo-2-methylphenyl)-4-methoxybenzenesulfonamide	99.21	2.47	Bisarylsulphonamides
6149306	3-chloro-4-[(5-chloro-2-methoxyphenyl)amino]-1-(2,3-dimethylphenyl)-1H-pyrrole-2,5-dione	99.60	2.39	Singleton
5301107*	N-(5-amino-9,10-dioxo-9,10-dihydro-1-anthracenyl)benzamide	100.00	3.27	Bisarylamides
5378429	6-chloro-2-methyl-N'-(4-methylbenzylidene)imidazo[1,2-a]pyridine-3-carbohydrazide	100.00	1.19	Hydrazones
5220809	3-[(2-methoxyphenyl)amino]-5-phenyl-2-cyclohexen-1-one	100.00	0.62	Bisarylamines
5249701	N'-(4-bromobenzylidene)-2,3-dihydro-1,4-benzodioxine-2-carbohydrazide	100.00	0.62	Hydrazones
6497060*	4-isopropoxy-N-({[4-(1-piperidinyl)phenyl]amino}carbonothioyl)benzamide	100.84	0.84	Phenylcarbamothioylbenzamides
5175570	2-methoxyethyl 5,6,7,8-tetrahydro[1]benzothieno[2,3-d][1,3]thiazol-2-ylcarbamate	101.07	2.23	Singleton
5783994	4-benzyl-N-(2,4-dimethoxybenzylidene)-1-piperazinamine	101.11	1.28	Hydrazones
5373649	3,4-dihydro-2(1H)-quinolinone phenylhydrazone hydrochloride	101.19	1.19	Hydrazones
5320101	2-[(2-methylbenzylidene)amino]benzamide	101.19	0.00	Benzylideneaminobenzamides

Supplementary Table 7. Selected Chemotypes Capable of Inhibiting DHODH. A single example of each unique chemotype from Supplementary Tables 5 and 6 that was capable of inhibiting DHODH to ≥40% activity at 10 μM.

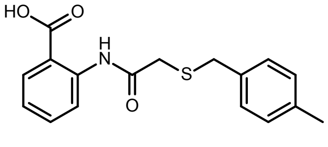

Bisarylamide (**ID 7723007**)

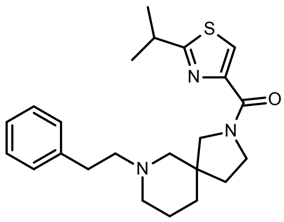

2-[(2-isopropyl-1,3-thiazol-4-yl)carbonyl]-7-(2-phenylethyl)-2,7-diazaspiro[4.5]decane (**ID 51157786**)

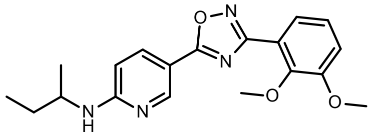

Aryloxadiazole (**ID 55410102**)

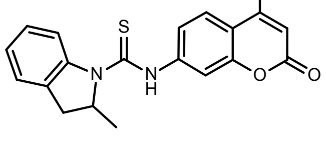

Chromen-2-one (**ID 7841649**)

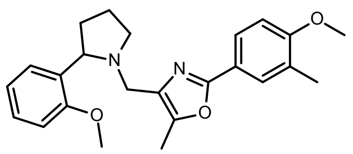

Aryloxazole (**ID 47870602**)

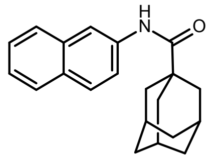

N-2-naphthyl-1-adamantanecarboxamide (**ID 5135537**)

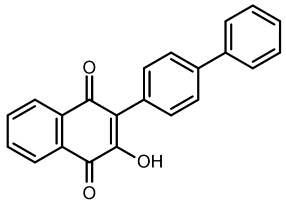

2-(4-biphenylyl)-3-hydroxynaphthoquinone (**ID 5248918**)

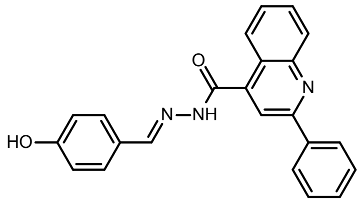

Hydrazone (**ID 5347717**)

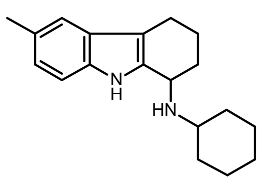

Tetrahydrocarbazole (**ID 5303009**)

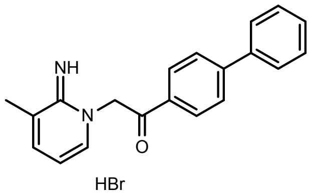

1-(4-biphenylyl)-2-(2-imino-3-methyl-1(2H)-pyridinyl)ethanone hydrobromide (**ID 5120580**)

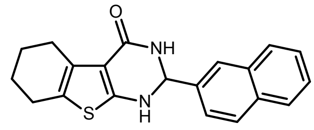

Thienopyrimidinone (**ID 5232020**)

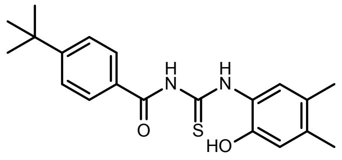

Phenylcarbamothioylbenzamide (**ID 6611243**)

Supplementary Table 8. List of antibodies used in this study.

Antigen	Source/Reference
hdm2 (human mdm2)	IF2, Calbiochem
p21	118. #SC-53870, Santa Cruz and see supplementary information[14].
p53	DO7 See supplementary information[15].<br>DO1 See supplementary information[16].
gapdh	# ab8245, Abcam
P-ATR (phospho Thr 1989)	# EVU001, KeraFAST
P-chk1 (phospho Ser 345)	# 2341, Cell Signaling
chk1	# SC-8408, Santa Cruz
P-ATM (phospho Ser 1981)	# ab81292, Abcam
ATM	# ab78, Abcam
α-tubulin	# T6199, Sigma
P-Ser15 p53 (phosphor Ser 15)	# 9284, Cell Signaling
γ-H2AX	# JBW301, Millipore
cdc6	# SC-9964, Santa Cruz
hdmx (human mdmx)	# A300-287 A, Bethyl Laboratories
nucleolin	# 22758, Abcam

Supplementary Table 9. DHODH and HZ05 crystallography. Data collection and refinement statistics from co-crystallisation of DHODH with (*R*)-HZ05.

Resolution (Å)	30-1.70 (1.74–1.70)
Wavelength (Å)	0.09795
Space group	$P3_221$
Unit cell (Å)	a=90.9, b=90.9, c=122.7
Completeness (%)	99.1 (86.1)
Redundancy	10.7 (7.6)
No. of observations / unique reflections	694626 (64865)
<I/σ(I)>	23.4 (4.3)
CC(1/2) (%)	99.9 (86.4)
R_{merge} (I) (%)	6.2 (46.5)
R_{cryst} (F) (%)	10.6
R_{free} (F) (%)	13.8
No. of non-hydrogen atoms	3320
No. of water molecules	257
rms deviations from ideal geometry: Bond lengths (Å)	0,021
Bond angles (deg)	2.3
Mean B-factor protein (Å^2)	25.4
Mean B-factor (solvent, Å^2)	36.8
Mean B-factor (ligand (*R*)- HZ05, Å^2)	20.6
Mean B-factor (other ligands, Å^2)	42.2

References to Supplementary Information

1. Guo, S. et al. Identification, synthesis, and pharmacological evaluation of tetrahydroindazole based ligands as novel antituberculosis agents. *J Med Chem* **53**, 649-59 (2010).
2. Hong, S.P. et al. Tricyclic thiazolopyrazole derivatives as metabotropic glutamate receptor 4 positive allosteric modulators. *J Med Chem* **54**, 5070-81 (2011).
3. Houston, J.B. Utility of in vitro drug metabolism data in predicting in vivo metabolic clearance. *Biochem Pharmacol* **47**, 1469-79 (1994).
4. Hubatsch, I., Ragnarsson, E.G. & Artursson, P. Determination of drug permeability and prediction of drug absorption in Caco-2 monolayers. *Nat Protoc* **2**, 2111-9 (2007).
5. Obach, R.S. Prediction of human clearance of twenty-nine drugs from hepatic microsomal intrinsic clearance data: An examination of in vitro half-life approach and nonspecific binding to microsomes. *Drug Metab Dispos* **27**, 1350-9 (1999).
6. Walse, B. et al. The structures of human dihydroorotate dehydrogenase with and without inhibitor reveal conformational flexibility in the inhibitor and substrate binding sites. *Biochemistry* **47**, 8929-36 (2008).
7. Murshudov, G.N. et al. REFMAC5 for the refinement of macromolecular crystal structures. *Acta Crystallogr D Biol Crystallogr* **67**, 355-67 (2011).
8. van Leeuwen, I.M. et al. Mechanism-specific signatures for small-molecule p53 activators. *Cell Cycle* **10**, 1590-8 (2011).
9. Bunz, F. et al. Requirement for p53 and p21 to sustain G2 arrest after DNA damage. *Science* **282**, 1497-501 (1998).
10. Kenzelmann Broz, D. et al. Global genomic profiling reveals an extensive p53-regulated autophagy program contributing to key p53 responses. *Genes Dev* **27**, 1016-31 (2013).
11. Fischer, M., Quaas, M., Steiner, L. & Engeland, K. The p53-p21-DREAM-CDE/CHR pathway regulates G2/M cell cycle genes. *Nucleic Acids Res* **44**, 164-74 (2016).
12. Chou, T.C. Theoretical basis, experimental design, and computerized simulation of synergism and antagonism in drug combination studies. *Pharmacol Rev* **58**, 621-81 (2006).
13. Lain, S. et al. Discovery, in vivo activity, and mechanism of action of a small-molecule p53 activator. *Cancer Cell* **13**, 454-63 (2008).
14. Fredersdorf, S., Milne, A.W., Hall, P.A. & Lu, X. Characterization of a panel of novel anti-p21Waf1/Cip1 monoclonal antibodies and immunochemical analysis of p21Waf1/Cip1 expression in normal human tissues. *Am J Pathol* **148**, 825-35 (1996).
15. Vojtesek, B., Bartek, J., Midgley, C.A. & Lane, D.P. An immunochemical analysis of the human nuclear phosphoprotein p53. New monoclonal antibodies and epitope mapping using recombinant p53. *J Immunol Methods* **151**, 237-44 (1992).
16. Bartkova, J. et al. Immunochemical analysis of the p53 oncoprotein in matched primary and metastatic human tumours. *Eur J Cancer* **29A**, 881-6 (1993).
