# Supplementary material for: A DHODH inhibitor increases p53 synthesis and enhances tumor cell killing by p53 degradation blockage
Source: Nat Commun. 2018 Mar 16;9:1107. doi: 10.1038/s41467-018-03441-3 (PMC5856786; doi:10.1038/s41467-018-03441-3)
Supplement: Supplementary file 3 — Description of Supplementary Files [file 41467_2018_3441_MOESM3_ESM.pdf]

Description of Supplementary Files

File Name: Supplementary Data 1
Description: The full RNAseq raw data as well as the processed data including the method of analysis.
